# Summarizing the effects of different exercise types in chronic neck pain – a systematic review and meta-analysis of systematic reviews

**DOI:** 10.1186/s12891-023-06930-9

**Published:** 2023-10-12

**Authors:** Eva Rasmussen-Barr, Marie Halvorsen, Tony Bohman, Carina Boström, Åsa Dedering, Roman P. Kuster, Christina B. Olsson, Graciela Rovner, Elena Tseli, Lena Nilsson-Wikmar, Wilhelmus Johannes Andreas Grooten

**Affiliations:** 1 Division of Physiotherapy, Department of Neurobiology, Care Sciences and Society, Karolinska Institutet, Alfred Nobels Allé 23, Huddinge, Sweden; 2 Department of Occupational Therapy and Physiotherapy, Women’s Health and Allied Health Professionals’ Theme, Karolinska University Hospital, Stockholm, Sweden; 3 School of Health and Welfare, Dalarna University, Falun, Sweden; 4 The Health and Medical Care Administration, Region Dalarna, Falun, Sweden; 5 Academic Primary Healthcare Centre, Region Stockholm, Stockholm, Sweden; 6 ACT Institutet Sweden, Research and Education, Gothenburg, Sweden

**Keywords:** Long-term pain, Physiotherapy, Training, Rehabilitation, Umbrella review

## Abstract

**Background:**

To date, no consensus exists as to whether one exercise type is more effective than another in chronic neck pain. This systematic review and meta-analysis of systematic reviews aimed to summarize the literature on the effect of various exercise types used in chronic neck pain and to assess the certainty of the evidence.

**Methods:**

We searched the databases Ovid MEDLINE, Embase, Cochrane Library, SportDiscus, and Web of Science (Core Collection) for systematic reviews and meta-analyses on adults between 18 and 70 years with chronic neck pain lasting ≥ 12 weeks which investigated the effects of exercises on pain and disability. The included reviews were grouped into motor control exercise (MCE), Pilates exercises, resistance training, traditional Chinese exercise (TCE), and yoga. Study quality was assessed with AMSTAR-2 and the level of certainty for the effects of the exercise through GRADE. A narrative analysis of the results was performed and in addition, meta-analyses when feasible.

**Results:**

Our database search resulted in 1,794 systematic reviews. We included 25 systematic reviews and meta-analyses including 17,321 participants (overlap not accounted for). The quality of the included reviews ranged from critically low to low (*n* = 13) to moderate to high (*n* = 12). We found low to high certainty of evidence that MCE, Pilates exercises, resistance training, TCE, and yoga have short-term positive effects on pain and that all exercise types except resistance training, show positive effects on disability compared to non-exercise controls. We found low to moderate certainty of evidence for conflicting results on pain and disability when the exercise types were compared to other exercise interventions in the short-term as well as in intermediate/long-term apart for yoga, as no long-term results were available.

**Conclusion:**

Overall, our findings show low to high certainty of evidence for positive effects on pain and disability of the various exercise types used in chronic neck pain compared to non-exercise interventions, at least in the short-term. Based on our results, no optimal exercise intervention for patients with chronic neck pain can be recommended, since no large differences between the exercise types were shown here. Because the quality of the included systematic reviews varied greatly, future systematic reviews need to increase their methodological quality.

**Trial registration:**

Prospero CRD42022336014.

**Supplementary Information:**

The online version contains supplementary material available at 10.1186/s12891-023-06930-9.

## Introduction

Musculoskeletal disorders are highly prevalent globally, leading to personal suffering and high socio-economical costs [1]. Neck pain, together with low back pain, is one of the most common musculoskeletal disorders related to years lived with disability according to the Global Burden of Disease studies [1]. The estimated one-year incidence of neck pain is around 20% – with a higher incidence reported among office and computer workers – and is reportedly higher in women [2–4]. Furthermore, between 30 to 50% of the adult population have experienced neck pain in the previous year, and a high percentage report recurrent pain [5]. Strong risk factors for developing neck pain or for developing recurrent neck pain include social determinants of health such as psycho-social factors rather than physical factors, such as high muscular tension, depressed mood, role conflict, and high job demand [6]. For acute or sub-acute neck pain to translate into chronic neck pain, non-modifiable factors have been suggested – including age, gender, and co-morbidity with other disorders – as well as modifiable factors such as psychological problems, sleep troubles, job stress, and work-related positions/posture [7–10].

Patients with neck or back pain have high levels of healthcare utilization both in primary and specialist healthcare [11]. Several treatments are offered, including pharmacological and non-pharmacological treatments such as electrotherapy [12], manual therapy [13], massage [14], and acupuncture [15], but the evidence for the effectiveness of these treatments varies [16]. Guideline-endorsed treatments for chronic neck pain include advice, education, and manual therapy as well as recommendations for physical exercise programs [17, 18]. Exercise is further suggested to be an intervention with minimal negative adverse effects [16] and seems to be a cost-effective treatment for chronic neck pain compared to treatments such as manual therapy or massage [19].

Various exercise types are used in the rehabilitation of chronic neck pain and are suggested as potentially beneficial, although the evidence for these effects is low and results are inconsistent [16, 20]. The exercise types are summarized in several systematic reviews reporting various effects for specific exercises such as motor control exercises [21], yoga [22], and Pilates [23] as well as strength and endurance training [20].

The stability of the neck is dependent on several deep and superficial muscles as well as on the posture of the neck and loads transferred via the arms [24, 25]. The exercise types used in chronic neck pain have different aims such as training of the deep neck flexors through motor control exercises, strength training of superficial muscles in the neck and shoulder girdle, or stabilization and endurance training aiming to keep the neck stable during loaded arm movements [26].

Our research group previously conducted a systematic review (SR) of SRs on the effect of various exercise types used in chronic low back pain and concluded that no exercise type seems to have more effect on pain and disability than any other [27]. A SR of SRs can help in summarizing the effect in a specific research area even if such a SR is itself dependent on the quality of the included SRs [28]. To date, there is no consensus if one exercise type is more effective than another in the treatment of chronic neck pain. Further, it can be of use for the therapist in their dialogue with the patient to decide on what exercises to choose and preferably based on the best evidence. The aim of this SR of SRs was therefore to summarize the literature on the effect of various exercise types used in chronic neck pain and to assess the certainty of the evidence.

## Methods

This study followed the PRISMA guidelines for systematic review [29] (Additional file 1). The method described in this study is the same as in our previous systematic reviews of systematic reviews on exercises used in chronic low back pain [27]. A protocol for the trial was registered in Prospero (CRD42022336014). No deviations were made from the protocol.

### Eligibility criteria

We included SRs and meta-analyses (MAs) in which a majority (> 75%) of the included original studies were randomized controlled trials (RCTs). We based the inclusion on PICO: population, intervention, comparator, and outcome (Additional file 2). We did not exclude any of the SRs or MAs in terms of language; treatment duration, frequency, or intensity; comparator intervention; follow-up time; or year of publication. Hereafter, all SRs (with or without MAs) will be referred to as SRs.

#### Patients

We included SRs mainly (> 75%) based on a working population aged 18 to 70 years, which defined their populations as suffering from chronic neck pain (defined as having neck pain for 12 weeks or more). The rationale to only include SRs with chronic neck pain was to gain a homogenous population [30].

#### Intervention

We included SRs in which the effect of any exercise therapy or training type was studied as the main (single) intervention. Exercise was defined as “a regimen or plan of physical activities designed and prescribed for specific therapeutic goals, with the purpose to restore normal musculoskeletal function or to reduce pain caused by diseases or injuries” [31].

#### Comparator

We did not set any limitations for comparator interventions.

#### Outcome

We included SRs that investigated pain and disability as outcomes in short-, intermediate-, or long-term follow-up. We defined the duration of follow-up as short-term (one day to three months), intermediate-term follow-up (three months up to, but not including, one year), and long-term follow-up (one year or longer) [32].

### Database search

We (authors ERB and WG) developed in collaboration with librarians at the Karolinska Institutet Library a comprehensive search strategy based on earlier published search strategies in Cochrane Reviews regarding exercise therapy and chronic neck pain in the following databases: Ovid MEDLINE, Embase, Cochrane Library (the Cochrane Database of Systematic Reviews), Web of Science (Core Collection), and SportDiscus. We combined search terms and MESH terms in a search strategy developed for Ovid MEDLINE and adapted this strategy for the other databases. For each search concept Medical Subject Headings (MeSH-terms) and free text terms were identified. SRs and MAs were considered in the database searches. Search strategies are presented in Additional file 3. After the original search was performed on 29 April 2022, the search was updated on 28 June 2023, using the methods described by Bramer et al. (2017) [33]. The data were then exported to Endnote (version 20). After removing all duplicates in Endnote using the methods described by Bramer et al. (2016) and comparing the DOIs, the papers were exported to Rayyan QCRI [34, 35]. All papers were alphabetically divided among five teams with two or three reviewers each. The reviewer pairs screened the titles and abstracts retrieved from the searches independently from each other and assessed these for eligibility against the predetermined inclusion criteria (PICO). At this stage of the process, regular reviewer meetings were held to reach a consensus. All titles and abstracts meeting the inclusion criteria were retrieved in full text. In each pair, both reviewers independently checked the full-text articles to assess their eligibility for the final inclusion in this review. Reasons for exclusion were noted in this stage, and if more than one reason for exclusion was available, the publication was excluded in PICO order, that is, a publication with wrong intervention, wrong publication type, and the wrong population was classified only as excluded based on population. We scrutinized the reference lists of the included SRs for additional potentially relevant publications.

### Overlap

Overlap was defined when the same original study was included in more than one of the included SRs [36]. We calculated the total overlap (original RCTs in our included SRs) for each type of exercise type independent of the outcome following the formula proposed by Pieper et al. [36]. We present the overlap with the percentage of corrected covered area (CCA). Interpretation of CCA: 0–5% = slight overlap, 6–10% = moderate overlap, 11–15% = high overlap, and > 15% = very high overlap.

### Assessment of the methodological quality of the included reviews

We conducted the assessment of the methodological quality using the recommended and updated tool AMSTAR-2 (A MeaSurement Tool to Assess systematic Reviews), which is considered valid and reliable when assessing SRs and MAs [37]. AMSTAR-2 has previously been used to assess the methodological quality in SRs of SRs [32, 38]. Our included SRs were assessed based on their score on AMSTAR-2 and thereafter assigned to one of four levels (critically low, low, moderate, and high), depending on the number of critical flaws and weaknesses as recommended by the designers of the tool [37]. Items 1, 4, 5, 6, 8, and 9 were classified as critical flaws, and all other items were classified as study weaknesses [37]. The two reviewers from each of the five pairs performed their assessments independently and compared them with each other. Disagreements in the assessments were handled in a consensus dialogue after comparing discrepancies between assessors and were discussed among the whole research group guided by ERB and WG.

### Data extraction and synthesis

One reviewer per pair extracted data from the included SRs, and the other reviewer from the same pair checked the extraction for accuracy. We extracted the data into a data extraction form adapted from a Cochrane form [29]. We extracted data primarily from the included SRs. If the data presented in the included SRs were in doubt, the original included RCTs were checked for accuracy. The results of each included SR were separated on the outcomes of pain and disability and on short-, intermediate-, and long-term follow-up.

The results were synthesized based on narrative and quantitative analyses. In the narrative analyses, the results were compared to a control intervention for between-group statistical significance. For each exercise type the outcome (pain/disability) and the follow-up time (short- and intermediate/long), the overall between-effects were classified into “positive effects”, indicating significant results in favor of the specific exercise type, “negative effects”, indicating significant results in favor of the control group, “no effects”, indicating no significant differences between the intervention and control groups, or “varying” effects, when different SRs showed different results, i.e. positive, negative or no results). The narrative analyses were performed separately for each exercise type and comparisons with non-exercising controls (usual care, education, etc.) and with exercising controls were made, as well as for short, respective intermediate/long follow-up periods.

Quantitative analysis using meta-analysis was also performed when at least two SRs provided aggregated data on the same intervention, the same type of control group (non-exercising or exercising), the same outcome (pain, disability), and the same follow-up time (short, intermediate/long-term). If one SR provided multiple results, these were pooled before entering the meta-analysis. Data required for the meta-analysis were extracted from the data presented in the included SRs. The software Review Manager [39] was used and Standardized Mean Differences (SMDs) were computed using a random effects model for each intervention. The generic inverse variance method was used, which permits a wide selection of data formats in the analyses [40]. For example, for SRs which reported Weighted Mean Differences (WMDs) or Pooled Mean Differences (PMDs), when necessary, the original data from the included RCTs were used to calculate an SMD for this specific SR before entering the meta-analysis. For every meta-analysis, measures of statistical heterogeneity (I^2^) were assessed. Funnel plots were used to assess potential publication bias. When two separate SRs presented the same data from the same original RCTs in their analysis, we chose to include only one of them to avoid double counting.

### Assessment of the certainty of the evidence

We used the GRADE approach to evaluate the certainty of evidence for each exercise type and each separate outcome [41]. In short, the first step of GRADE is to choose a starting point for the level of evidence. Because our included SRs only comprised RCTs, we decided to start at the highest level. We thereafter lowered the certainty of evidence by appraising the potential risk of bias due to study limitations (high risk of bias based on the AMSTAR ratings), inconsistency in results (heterogeneity), imprecision (large confidence intervals), indirectness (generalizability of population and interventions), and publication bias (funnel plots). The certainty of evidence was increased if large effects were presented in the SRs or if a “dose–response” was seen based on the reports of the SRs. In this way, we express our findings together with the certainty of evidence in the results using four levels of evidence: “high” (+ +  + +), “moderate” (+ + +), “low” (+ +), or “very low” ( +) [41].

## Results

### Search results

**Fig. 1:**
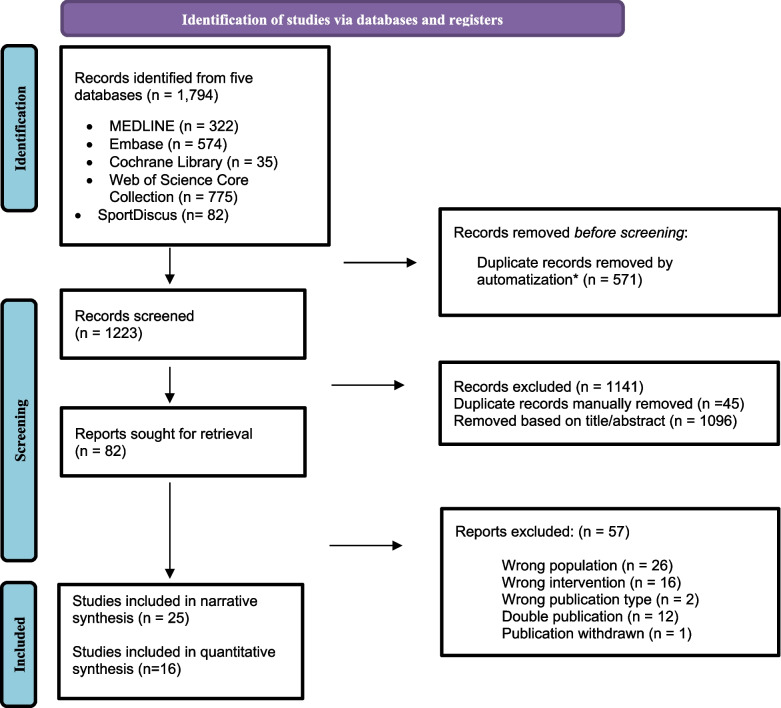
PRISMA chart for eligible study selection process. *Consider, if feasible to do so, reporting the number of records identified from each database or register searched (rather than the total number across all databases/registers)

The search results are summarized in Fig. 1. The literature search returned a total of 1,794 records. Following removal based on duplicates, a review of the titles and abstracts (*n* = 1,223) was performed, and 82 full texts were screened. Automatic de-duplication was based on the method described by Bramer et al. [35]. After checking against our inclusion and exclusion criteria, we included 25 SRs in the final review, which included a total of 221 randomized controlled trials (RCTs) in which 17,321participants were included (overlap not accounted for). Taking overlap into consideration, a total of 125 (original) studies were included in the 25 SRs. All included SRs were in English. A list of excluded SRs and reasons for exclusion is included in Additional file 4.

### Study characteristics

Our included SRs were published from 2010 to 2023. The majority (80%; 20 out of 25) were MAs, and most of the included patients were defined as having chronic neck pain for at least 12 weeks (Table 1).

**Table 1:** Description of the included systematic reviews; the number of original studies included, population, exercise intervention, and controls

**Motor Control exercises (inclusive Pillar exercises)**			
**Authors (year)**	**Population** Number of subjects, Chronic Neck Pain definition, pain duration, sex, age	**Intervention** Description, Training period	**Control** Description, Training period
Ferro Moura Franco, et al. (2021) [42]	**Number of subjects:** 182 **Definition:** Chronic idiopathic neck pain **Duration:** ≥ 12 wks **% Women**: 73% **Age:** Mean 38 yrs	**Description:** Motor Control Exercises **Training period:** 4–12 wks. 2–3 sessions/wk. 20–40 min/session	**Description:** Muscle strengthening/muscle endurance training **Training period:** 4–12 wks
Garzonio, et al. (2022) [21]	**Number of subjects:** 717 **Definition:** Chronic nonspecific neck pain **Duration:** ≥ 3 mo to 1 yr **% Women**: Between 40–100% **Age:** Mean 32 yrs	**Description:** Motor Control Exercises **Training period:** Varied from 4–12 wks; 1–7 times/wk	**Description:** Active exercises (e.g., aerobic, high load, conventional), other types of physical intervention (e.g., physical therapies, manual therapy, education, and pharmacological or surgical treatments, or passive treatment, e.g., placebo or waiting list, multimodal conventional treatments **Training period:** 4–12 wks, 1–7 times/wk
Hanney, et al. (2010) [43]	**Number of subjects:** 132 **Definition:** Adults with neck pain **Duration:** > 3 mo **% Women**: NR (1 study only women) **Age:** 18–70 yrs	**Description:** Motor Control Exercises **Training period:** 1–7 wks, 1–2 sessions/wk. Home exercises 30 min/session	**Description:** Muscle strengthening/muscle endurance training or non-specific strengthening training **Training period:** 4–7 wks
Martin-Gomez, et al. (2019) [44]	**Number of subjects:** 423 **Definition:** Adults with non-specific neck chronic neck pain **Duration:** ≥ 3 mo **% Women**: NR: (4 studies only women) **Age:** NR	**Description:** Motor Control Exercises through craniocervical flexion with pressure bio-feed-back unit **Training period:** 1–10 wks, 1–3 sessions/wk, 30–45 min/session	**Description:** Strengthening exercises neck flexors, proprioception exercises, active range of motion exercises, passive mobilization, wait and see **Training period:** NR
Mueller et al. (2023) [45]	**Number of subjects:** 1166 **Definition:** Chronic non-specific neck pain or neck pain **Duration:** ≥ 12 wks **% Women**: 62% (18 studies), in 3 studies NR **Age:** Mean 36 yrs	**Description:** Motor control exercise aimed to increase the control and coordination of deep cervical musculature or increase the proprioceptive control of head and neck movements **Training period:** The intervention period ranged between 1 and 24 weeks. Training frequency ranged from 2 to 14 sessions per week with 5–50 min/session	**Description:** Strength training, home exercises/digital video instructions, postural corrections, and lectures on promoting general health **Training period:** Same as the intervention group
Price, et al. (2020) [26] **Motor Control Exercises**	**Number of subjects:** 485 **Definition:** Non-specific neck pain **Duration:** > 3 mo **% Women:** NR (2 studies), range 40–100% (8 studies) **Age:** Mean (range) 38 (20–45) yrs	**Description:** Motor Control Exercises through craniocervical flexion with/without pressure biofeedback unit **Training period:** 1–12 wks, 1–7 sessions/wk	**Description:** Endurance training, pillar exercises active range of motion exercises, passive mobilization **Training period:** 1–12 weeks, 1–7 sessions/wk
Price, et al. (2020) [26] **Pillar Exercises**	**Number of subjects:** 263 **Definition:** Non-specific neck pain **Duration:** > 3 mo **% Women:** one study only female, one study 89% **Age:** Mean (range) 38 (25–51) yrs	**Description:** Pillar Exercises are intended to develop the ability of the spine to maintain a neutral position. Cervical isometric flexion/ extension/ rotation/ lateral flexion using the hand as resistance/ pulley system/ resistance bands; Cervical isometric flexion against gravity in sitting **Training period:** 4–8 wks, 2–7 sessions/wk	**Description:** Education, Motor Control Exercises, usual care **Training period:** 6 wks, 3 sessions/wk
Tsiringakis, et al. (2020) [46]	**Number of subjects:** 730 **Definition:** Chronic neck pain **Duration:** > 3 mo **% Women**: 42% (4 studies only women) **Age:** Mean 36 yrs	**Description:** Motor Control Exercises with pressure biofeedback unit **Training period:** 4–12 wks	**Description:** At least one of the control groups received no further intervention, a placebo intervention or another passive or active intervention without a pressure feedback device **Training period:** 4–12 wks
Villanueva-Ruiz, et al. (2022) [47]	**Number of subjects:** 468 **Definition:** Nonspecific neck pain **Duration:** ≥ 3 mo **% Women**: 63% **Age:** Mean 36 yrs	**Description:** Specific neck exercises (CFF) targeting deep cervical flexors and extensors with slow and controlled CCF using biofeedback unit **Training period**: ≥ 4 wks ≥ 6 mo, 30–45 min/session	**Description:** Alternative exercise intervention used strengthening, and/or endurance exercises as a sole exercise intervention **Training period:** ≥ 4 wks < 6 mo
**Pilates**			
**Authors (year)**	**Population** Number of subjects, Chronic Neck Pain definition, pain duration, sex, age	**Intervention** Description, Training period	**Control** Description, Training period
Martini et al. (2022) [23]	**Number of subjects:** 224 **Definition:** Neck pain with or without radicular symptoms **Duration:** 3 studies > 3mo, 1 study NR **% Women**: 82% **Age:** Range 23–61 yrs	**Description:** Different interventions based on the Pilates method using a Pilates mat and/or Pilates devices. One study reported supervised exercises and the other N.R **Training period:** 1–3 sessions/wk, for 8–12 wks	**Description:** Yoga, isometric exercises, motor control exercises and standard pharmacological treatment **Training period:** No intervention at all up to 8–10 weeks
**Resistance training**			
**Authors (year)**	**Population** Number of subjects, Chronic Neck Pain definition, pain duration, sex, age	**Intervention** Description, Training period	**Control** Description, Training period
Bertozzi, et al. (2013) [48]	**Number of subjects:** 1063 **Definition:** Chronic non-specific neck pain incl. trapezius myalgia **Duration:** > 3 mo **% Women**: 90% **Age:** Mean (Range) 39 (29–45) yrs	**Description:** Therapeutic exercise performed at home or the gym, including warm-ups, isometric and dynamic strengthening, coordination, stretching, deep neck muscle activation, and postural exercise, targeting muscles of the neck, shoulder, and upper extremities **Training period:** 1–28 sessions/wk for 10–60 min, for 2–52 wks (12–156 sessions in total)	**Description:** No intervention, partially including education or counseling, stretching, with medication as usual **Training period:** No intervention at all up to 3 sessions/wk for 52 wks
Cheng, et al. (2015) [49]	**Number of subjects:** 876 **Definition:** Chronic non-specific neck pain incl. upper-trapezius pain **Duration:** > 3 mo **% Women**: NR **Age:** Mean 37.9—45.6 yrs	**Description:** Exercise training for muscle strength and endurance for neck and shoulder including elastic bands and dumbbells, combined with traction and whole-body exercises, partially including relaxation and coordination **Training period:** 2–3 sessions/wk for 45–60 min, for 10–12 wks (short-term) and 1 yr (long-term)	**Description:** Not engaging in any exercise intervention, partially including stretching, education, and stress management **Training period:** No intervention at all or 1–3 sessions/wk for 20–120 min, for 10 wks—1 yr
Louw, et al. (2017) [50]	**Number of subjects:** 2075 **Definition:** Neck pain **Duration:** 3 mo (6 studies) to 6 mo (2 studies) **% Woman:** 77% **Age:** Mean 34.2 to 49 yrs	**Description:** Different types of progressive dynamic resistance exercises for neck and shoulder muscles **Training period:** 3–9 sessions/wk for 7–30 min, for 10 wks to 12 mo	**Description:** Health promotion activities (ergonomics, stress management, information to maintain regular physical activities, stretching, aerobic exercise) or no exercise **Training period:** NR
Mueller et al. (2023) [45]	**Number of subjects:** 1958 **Definition:** Chronic non-specific neck pain or neck pain **Duration: > **3 mo (20 studies) NR (1 study), non-traumatic (1 study), and NP intensity ≥ 3 (0–9 scale) last month (1 study) **% Woman:** 80% (20 studies). In 3 studies NR **Age:** Mean 36.4 (SD 7.1) yrs and in 1 study NR	**Description:** Exercises that aim to increase the strength, power, or endurance of the cervical- and scapula musculature **Training period:** 1**–7** sessions/wk for 7–60 min (in 6 studies NR), for 4 to 12 wks (17 studies), 14 sessions twice/day for 10–20 min for 6 wks (1 study), 14 sessions for 10–25 min during 6 wks (2 studies), every day for 25–30 min for 12 wks (1 study), session/wk NR (2 studies)	**Description:** Different activities (ergonomics, information to maintain regular physical activities, home exercises with digital video instructions) waiting list, exercise and spinal manipulation, craniocervical flexion training, deep cervical flexion training, scapula training, neck- and scapula stabilization training or not described **Training period:** Same as for the intervention group
Ouellet, et al. (2021) [51]	**Number of subjects:** 209 **Definition:** Persistent or chronic neck pain **Duration:** > 30 days (2 studies), > 12 wks (2 studies), > 6 mo (1 study) **% Women:** In 3 studies only women (27%) in 2 not reported **Age:** Mean age between 41–52 yrs (4 studies) and 76 yrs (1 study)	**Description:** Region-specific exercises: Specific supervised strength training for neck and shoulders (1 study) Strengthening of neck and Core, Proprioceptive exercises, Dynamic mobilization (1 study) Specific resisted exercises aimed at the neck/shoulder region (1 study) Standardized program including active movements, strengthening, and flexibility exercises (1 study) **Training period:** 10–12 wks	**Description:** General exercises: Aerobic; Monark bicycle ergometer (1 study), Tai Chi (1 study), Aerobic; Supervised walking program (1 study), Qigong (1 study) **Training period:** 10–12 wks
Seo, et al. (2020) [52]	**Number of subjects:** 122 **Definition:** Chronic pain in the cervical and shoulder region **Duration:** 3–6 mo **% Women**: NR **Age:** NR	**Description:** Scapular stabilization exercises are most often implemented as strengthening exercises targeting the trapezius and serratus anterior muscles. The exercise types were varied substantially **Training period:** 3–7 sessions/wk for 20–30 min, for 4–10 wks (12–42 sessions in total)	**Description:** NR **Training period:** NR
Price, et al. (2020) [26] **Resistance Training (2 studies)**	**Number of subjects:** 186 **Definition:** Non-specific neck pain excluding specific pathologies **Duration:** ≥ 3 mo **% Women:** NR **Age:** 18–70 yrs	**Description:** Upper limb Resistance Exercises (ULRE) such as triceps and shoulder press, biceps curls, pullovers, overhead and chest press, punches, rotations **Training period**: 3 sessions/wk with 2 or 3 sets of 12 repetitions, for 6–10 wks	**Description:** No treatment or education and stress reduction **Training period:** 1 session/wk for 10 wks
Yang, et al. (2022) [53]	**Number of subjects:** 1891 **Definition:** Chronic **Duration:** ≥ 3 mo **% Women:** NR **Age:** mean age ranged between 16–58 yrs	**Description:** Isometric training refers to increasing muscle tension to fight against a fixed resistance exercise **Training period**: 2–8 wks; 6–24 sessions	**Description:** non-isometric exercises or no training intervention: neuroregulations, exercise therapy, lectures, conventional therapy, TCM therapy **Training period:** 2–8 wks; 6–24 sessions
**Traditional Chinese exercises with a focus on Qigong and Tai Chi**			
**Authors (year)**	**Population** Number of subjects, Chronic Neck Pain definition, pain duration, sex, age	**Intervention** Description, Training period	**Control** Definition, Training period
Bai, et al. (2015) [54]	**Number of subjects:** 161 **Definition:** Recurrent neck pain > 6 mo (1 study). The minimum of neck pain between 6 mo and 5 yrs (1 study) **Duration:** Mean 3.17 yrs, (1 Study), mean 19 (SD 14.9) yrs (1 study) **% Women:** 87.7%—95% **Age:** Mean 45.6 (SD 10.17) (1 study), mean 76 (SD 8) (1 study) yrs	**Description:** Neiyanggong Qigong (1 study) and Dantian Qigong (1 study) including neck exercises, shoulder exercises and moving (closing) exercises, and breathing exercises **Training period:** 18–24 sessions over 6 mo (1–2 sessions/wk), 45–90 min/session. Supervised 0–3 mo, 3–6 mo self-training	**Description:** Exercise therapy included warming-up with a softball, main exercises with theraband, mainly neck exercises, and closing exercise and active cervical rotations, strength and flexibility exercises **Training period:** Exercise therapy was supervised 18–24 sessions over 6 mo, 2–3 sessions/wk, 45 min/session. Self-training between month 3 and 6 (1 study) The waitlist group received no intervention but was offered treatment of their choice after 6 months (1 study)
Ferro Moura Franco, et al. (2020) [42]	**Number of subjects:** 355 **Definition:** Chronic idiopathic neck pain **Duration:** range 3 mo to > 10 yrs **% Women**: 81% **Age:** Mean (SD) 54 (9.2) yrs	**Description:** All sessions supervised. Tai Chi based on Yang style (1 study). Qigong, including different methods; Dantian method (1 study), Neiyanggong method (1 study) and Biyun Medical Qigong (1 study). All with different combinations of body postures, movement, breathing, meditation, relaxation and sometimes self-massage **Training period:** 12–26 wks, 1–2 sessions/wk (12–24 sessions in total), 45–90 min/session	**Definition:** All sessions supervised, some combined with home exercise. Exercise therapy for neck pain, individually tailored programs combining e.g. mobility, proprioceptive exercises, stretching, strengthening exercises and endurance training **Training period:** 12–26 wks, 1–2 sessions/wk (12–24 sessions in total), 45–75 min/session
Girard, et al. (2019) [55]	**Number of subjects:** 362 **Definition:** Chronic neck pain **Duration:** > 3 mo **% Women**: 84% **Age:** Mean (SD) 55.3 (6.3) yrs	**Description:** Qigong, including different methods; Dantian method (1 study), Nestudyiyanggong method (1 study) and Biyun Medical Qigong (1 study). All with different combinations of body postures, movement, breathing, meditation, relaxation and sometimes self-massage **Training period:** 12–24 wks, 1–2 sessions/wk (12–24 sessions in total), 45–90 min/session	**Definition:** Standardized exercise therapy for computer and workplace-related neck pain and individually tailored exercise programs combining e.g. mobility, stretching, strengthening exercises and endurance training Watilist control **Training period:** 12–24 wks, 1–2 sessions/wk (12–24 sessions in total), 45–90 min/session
Gross, et al. (2016) [32]	**Number of subjects:** 239 **Definition:** Chronic mechanical neck disorders **Duration:** NR **% Woman:** NR **Age:** only reported in elderly adults	**Description:** Qigong: Neck and shoulder exercises including relaxation of mind and body, conscious breathing and movement exercises of the hip, legs, shoulders, arms and head. home exercise with a manual. ROM, use of softball, strengthening using a theraband; flexibility exercise **Training period:** 18–24 sessions, 45–90 min/session	**Description:** No intervention /waitlist control
**Kong et al. (2022) (57)**	**Number of subjects:** 468 **Definition:** Neck pain **Duration:** NR **% Women**: NR **Age:** Mean 54.8 yrs	**Description**: Traditional Chinese mind and body exercises. Qigong with body postures, deep meditation, purposeful breathing, relaxation and self-massage (1 study), Tai Chi with relaxing music and breathing exercises (2 studies). Traditional Chinese exercises, fitness Qigong (1 study) and Five-animal exercises (1 study) **Training period:** 10–24 wks., 1–6 sessions/wk (12–60 sessions in total), 45–90 min/session	**Description:** Exercise therapy (3 studies). Passive treatment; Tuina (1 study), Traction (1 study) Waitlist control (3 studies) **Training period:** 12–24 wks, 1–2 sessions/wk (10–24 sessions in total), 45–75 min/session **Treatment period (passive):** 10-wks, 3–4 sessions/wk (30–40 sessions in total), 20–60 min/session
Xie, et al. (2021) [56]	**Number of subjects:** 605 **Definition:** Neck pain (4 study chronic neck pain) **Duration:** NR **% Women**: 70.5% **Age:** Mean (SD) 51.7 (12.7) yrs	**Description**: Traditional Chinese mind and body exercises. Qigong with body postures, deep meditation, purposeful breathing, relaxation and self-massage (3 trials), Tai Chi with relaxing music and breathing exercises (1 trial) and the 12-words-for-life-nurturing exercise (1 trial) with massaging acupoints or a part of the body, deep breathing, and regulating body postures **Training period:** 3–6 mo, 1–2 sessions/wk (12–24 sessions in total), 45–90 min/session	**Description:** Exercise therapy. Modern rehabilitation treatments, including cervical manipulation, mobility, stretching, strengthening exercises, endurance training, other modern exercise therapy or minimal intervention **Training period:** 12–26 wks, 1–2 sessions/wk (10–24 sessions in total), 45–75 min/session
Yuan, et al. (2015) [57]	**Number of subjects:** 362 **Definition:** chronic neck pain **Duration:** > 3 mo **% Women:** 84% **Age:** Mean (SD) 55.3 (6.3) yrs	**Description:** Qigong, including different methods; Dantian method (1 study), Nestudyiyanggong method (1 study) and Biyun Medical Qigong (1 study). All with different combinations of body postures, movement, breathing, meditation, relaxation and sometimes self-massage	**Description:** Standardized exercise therapy for computer and workplace-related neck pain and individually tailored exercise programs combining e.g. mobility, stretching, strengthening exercises and endurance training Waitlist control
De Zoete, et al. (2020) [58]	**Qigong** (2 studies) **Number of subjects:** 239 **Definition:** Chronic idiopathic neck pain (neck pain lasting longer than 12 wks) **Duration:** NR **% Women**: 82% **Age:** Mean 44 yrs (1 study) and 76 yrs (1 study) **Tai-Chi** (1 study) **Number of subjects:** 114 **Definition:** NR **Duration:** NR **% Women: 80**% **Age:** mean 49.4 yrs	**Qigong:** **Description:** soft, whole-body movements with a focus on relaxation, posture and breathing **Training dose:** 12 wks; 2 sessions/wk; 45–60 min/session **Tai-Chi:** **Description:** soft, whole-body movements with a focus on relaxation, posture and breathing **Training period:** 12 wks	**Qigong** **Description:** Neck-specific exercises and waiting list control **Training dose:** 12 wks **Tai-Chi** **Description:** Waiting list control, neck-specific exercises **Training period:** 12 wks
**Yoga**			
**Authors (year)**	**Population** Number of subjects, Chronic Neck Pain definition, pain duration, sex, age	**Intervention** Description, Training period	**Control** Description, Training period
Cramer, et al. (2017) [59]	**Number of subjects:** 188 **Definition:** Chronic non-specific neck pain/disability defined as more than 12 wks **Duration:** NR **% Women**: 82.4% **Age:** Mean (SD) 45.8 (12.3) yrs	**Description:** Studies on yoga interventions including at least one of the following: Physical activity, breath control, meditation, and/or lifestyle advice (based on yoga theory and/or traditional yoga practices) No restrictions were made regarding yoga tradition, length, frequency, or duration of the program. Studies on multimodal interventions that include yoga among others were excluded Iyengar yoga (physical postures) (2 studies), Yogic mind sound resonance technique (meditation) (1 study) **Training period:** between 10 days to 9 wks (median length: 9 wks). Iyengar yoga for 9 wks 1 session/wk 90 min/session (2 studies). Yogic mind sound resonance technique for 10 days 20 min/session daily + physiotherapy 10 days 30 min/session daily (1 study)	**Description:** Usual care, self-care information, supine rest + physiotherapy **Training period:** From 10 days to 9 wks Usual care, self-care information 9 weeks (2 studies), Supine rest 10 days, 20 min daily + physiotherapy 10 days, 30 min daily (1 study)
Kim, et al. (2016) [60]	**Number of subjects:** 184 **Definition:** Chronic non-specific neck pain defined as a duration longer than 3 mo Neck pain intensity at least 40 mm on a 100-mm visual analog scale (VAS) or ≥ 3/10 on the numeric pain rating scale (NRS) **Duration:** NR **% Women**: 85.9% **Age:** Means ranged between 47.8 and 55.6 yrs	**Description**: Iyengar program (poses to lengthen and strengthen muscles in the neck and shoulders and to improve stability, flexibility, alignment, and mobility in muscles, joints, and tendons) (2 studies) Yoga program (comprised of regulated breathing and poses to improve alignment, strength, flexibility, and relaxation) (1 study) **Training period:** In Iyengar yoga, participants practiced 90 min/day, 1/wk for 9 wks (2 studies). Yoga for 60 min/day, 5 days/wk for 3 mo (1 study)	**Description:** Exercise 10–15 min/day self-care manual **Training. period:** wks (2 studies), no control group (1 study)
Li, et al. (2019) [22]	**Number of subjects:** 686 **Definition:** Persistent neck pain or severe discomfort in the neck > 3 mo **Duration:** NR **% Women**: NR **Age:** 18–55 with means (SD) between 20.8 (1.2) and 55.6 (9.0) yrs	**Description:** Yoga intervention, including both exercise-based and meditation-based **Training period: P**rogram length ranging from 10 days to 12 wks	**Description:** other therapies except yoga (e.g., exercise, Pilates, usual care, etc.) **Training period:** 4–15 wks, 1–3 sessions/wk (12–45 sessions in total), 45–60 min/session
De Zoete, et al. (2020) [58]	**Number of subjects:** 188 **Definition:** Idiopathic neck pain **Duration:** NR **% Women:** 84% **Age:** 18–60 with means between 39.7 and 47.9 yrs	**Description:** NR **Training period:** 6–9 wks	**Description:** Self-care manual including neck-specific exercises (2 studies) Standard physiotherapy (including isometric neck exercises, and electrophysical elements) (1 study)

*Abbreviations*: *min* minutes, *mo* months, *NR* Not reported, *yr* year, *yrs* years, *wk* week, *wks* weeks, *SD* Standard deviation, *TCM* Traditional Chinese Medicine

The 25 included SRs were grouped into five exercise types: a) motor control exercise (MCE) with craniocervical flexion and including Pillar exercises, b) Pilates exercises, c) resistance training, d) traditional Chinese exercise (TCE) such as Tai Chi and Qigong, and e) yoga. A description of the exercise types is presented in Table 2. In four SRs [26, 42, 45, 58] several exercise types were studied and were reported for each exercise type studied separately (Table 5). All but one of the included SRs [43] reported effects on pain, and five did not report effects on disability [21, 26, 42, 54, 58]. In the short-term perspective, some SRs diverted from our definition of < 12 weeks and defined short-term as up to 24 weeks [42, 45, 50].

**Table 2:** Description of the exercise types

Type of exercise	Description	References
Motor Control Exercises, including pillar exercises	Motor control exercises are defined as training of the deep neck muscles mostly using a craniocervical flexion hold without a bio-pressure feedback device Pillar exercises are defined as exercises intended to develop the ability of the spine to maintain a neutral position while giving resistance via pulleys, elastic bands, or by giving manual resistance to the head [26]	[21, 26, 42–47]
Pilates	Pilates exercises follow the traditional Pilates principles, such as centering, concentration, control, precision, flow, and breathing	[23]
Resistance training	Resistance training is any exercise that causes the muscle to contract against an external resistance to improve strength, power, endurance, and/or hypertrophy. The external resistance can be dumbbells, resistance bands, or the own body weight	[26, 45, 48–52]
Traditional Chinese exercises with a focus on Qigong and Tai-Chi	Traditional Chinese exercises include Traditional Chinese Mind and Body Exercise (TCMBE) which is a rehabilitation modality that has been used for neck pain by rehabilitation professionals. TCMBE was developed in China and includes several practices, such as Qigong, Tai-chi, and the 12-words-for-life-nurturing exercise. TCMBE has a variety of subsets, each of which has a unique action, and those subsets have common characteristics that integrate with holistic body concepts emphasizing the integration of body posture, breathing patterns, and mind adjustments to achieve beneficial effects on both mental and physical well-being	[32, 42, 54–58, 61]
Yoga	Yoga combines physical postures (asana), breathing techniques (pranayama), and meditation (dyana) to promote physical and mental well-being. There are a variety of different yoga styles focusing on these above-mentioned areas in a particular way	[22, 58–60]

### Quality of the included SRs

Based on the AMSTAR-2 ratings, we found five SRs with high quality [23, 26, 42, 51, 59], seven SRs with moderate quality [21, 32, 44–47, 57], eight SRs with low quality [22, 43, 48, 50, 53, 54, 56, 61], and five SRs with critically low quality [49, 52, 55, 58, 60]. The AMSTAR-2 ratings for all included publications are presented in Table 3. Of the six items that were identified as critical, most studies fulfilled these criteria, except for item 4 “Did the authors use a comprehensive literature search strategy?”, where only 13 out the of 25 SRs scored a “yes”. Concerning the remaining items, many studies lacked reporting on item 10 funding of the included studies (*n* = 23), and item 7 “Did not (or partially did not) include a list of excluded studies” (*n* = 20), and item 2 “Did not establish a protocol before the review” (*n* = 10).

**Table 3:** Summary of methodological quality assessment of included studies using AMSTAR-2

**Authors**	**1**	2	3	**4**	**5**	**6**	7	**8**	**9**	10	11	12	13	14	15	16	**Flaws** **(n)**	**Weakness** **(n)**	**Quality**
Bai et al. (2015) [54]	**Y**	N	Y	**PY**	**Y**	**Y**	N	**PY**	**Y**	N	Y	N	Y	N	Y	Y	**2 Partial**	**6**	**Low**
Bertozzi, et al. (2013) [48]	**Y**	N	N	**PY**	**Y**	**Y**	N	**Y**	**Y**	N	N	N	Y	Y	Y	N	**1 Partial**	**6**	**Low**
Cheng, et al. (2015) [49]	**Y**	N	N	**N**	**N**	**N**	N	**PY**	**Y**	N	N	N	N	N	N	N	**3** **1 Partial**	**10**	**Critically Low**
Cramer, et al. (2017) [59]	**Y**	Y	Y	**PY**	**Y**	**Y**	Y	**Y**	**Y**	N	Y	Y	Y	Y	Y	Y	**1 Partial**	**1**	**High**
Ferro Moura Franco, et al. (2020) [42]	**Y**	Y	Y	**Y**	**Y**	**Y**	N	**Y**	**Y**	N	Y	Y	Y	Y	Y	Y	**0**	**2**	**High**
Garzonio, et al. (2022) [21]	**Y**	Y	Y	**Y**	**Y**	**Y**	PY	**Y**	**Y**	N	Y	Y	Y	Y	Y	N	**0**	**2** **1 Partial**	**Moderate**
Girard, et al. (2019) [55]	**Y**	N	Y	**PY**	**Y**	**N**	N	**PY**	**PY**	N	N	N	N	N	N	Y	**1** **3 Partial**	**8**	**Critically low**
Gross, et al. (2016) [32]	**Y**	Y	Y	**Y**	**Y**	**Y**	Y	**PY**	**Y**	N	Y	N	Y	Y	Y	N	**1 Partial**	**3**	**Moderate**
Haney, et al. (2010) [43]	**PY**	N	Y	**Y**	**Y**	**N**	N	**Y**	**Y**	N	N	N	N	Y	-	N	**1** **1 Partial**	**7**	**Low**
Kim, et al. (2016) [60]	**Y**	PY	N	**PY**	**N**	**N**	N	**PY**	**Y**	N	N	N	Y	N	N	N	**2** **2 Partial**	**8**	**Critically low**
Kong et al. (2022) [61]	**Y**	Y	Y	**Y**	**Y**	**Y**	N	**PY**	**Y**	N	N	N	N	N	Y	Y	**1 Partial**	**6**	**Low**
Li, et al. (2019) [22]	**Y**	Y	N	**PY**	**Y**	**Y**	N	**PY**	**Y**	N	Y	N	Y	Y	N	Y	**2 Partial**	**5 low**	**Low**
Louw, et al. (2017) [50]	**Y**	N	N	**Y**	**Y**	**Y**	N	**Y**	**Y**	N	Y	N	PY	Y	N	Y	**0**	**6** **1 Partial**	**Low**
Mueller et al. (2023) [45]	**Y**	Y	N	**Y**	**Y**	**Y**	PY	**Y**	**Y**	N	Y	Y	N	N	Y	Y	0	**1 Partial**	**Moderate**
Martinez-Gomez, et al. (2019) [44]	**Y**	Y	Y	**Y**	**Y**	**Y**	PY	**Y**	**Y**	N	Y	N	N	Y	Y	Y	**0**	**3** **1 Partial**	**Moderate**
Martini et al. (2022) [23]	**Y**	**Y**	Y	**Y**	**Y**	**Y**	Y	**Y**	**Y**	Y	Y	Y	Y	Y	Y	Y	**0**	**0**	**High**
Ouellet, et al. (2021) [51]	**Y**	Y	Y	**Y**	**Y**	**Y**	Y	**Y**	**Y**	N	Y	Y	Y	Y	Y	N	**0**	**2**	**High**
Price, et al. (2020) [26]	**Y**	Y	Y	**Y**	**Y**	**Y**	Y	**Y**	**Y**	Y	Y	N	Y	Y	N	Y	**0**	**2**	**High**
Seo, et al. (2020) [52]	**N**	N	N	**N**	**Y**	**N**	N	**N**	**Y**	N	N	N	N	N	N	Y	**4**	**9**	**Critically Low**
Tsiringakis, et al. (2020) [46]	**Y**	N	Y	**Y**	**Y**	**Y**	PY	**Y**	**Y**	N	Y	N	Y	Y	Y	Y	**0**	**3** **1 Partial**	**Moderate**
Villanueva- Ruiz, et al. (2022) [47]	**Y**	Y	Y	**PY**	**Y**	**Y**	N	**Y**	**Y**	N	Y	Y	Y	Y	Y	Y	**1 Partial**	**2**	**Moderate**
Xie, et al. (2021) [56]	**Y**	Y	Y	**PY**	**Y**	**Y**	N	**PY**	**Y**	N	Y	N	Y	Y	N	Y	**2 Partial**	**4**	**Low**
Yang, et al. (2022) [53]	**Y**	N	N	**N**	**Y**	**Y**	N	**PY**	**Y**	N	Y	Y	Y	Y	Y	Y	**1**	**4**	**Low**
Yuan, et al. (2015) [57]	**Y**	N	Y	**Y**	**Y**	**Y**	N	**Y**	**Y**	N	Y	N	Y	Y	Y	Y	**0**	**3**	**Moderate**
de Zoete, et al. (2020) [58]	**Y**	PY	Y	**PY**	**Y**	**Y**	N	**N**	**Y**	N	N	N	Y	N	N	Y	**1** **1 Partial**	**5**	**Critically low**

The items marked in bold are in this study considered as critical flaws, while the non-marked items are considered as weaknesses

AMSTAR-2 Criteria: 1. Did the research questions and inclusion criteria for the review include the components of PICO? 2. Did the report of the review contain an explicit statement that the review methods were established prior to the conduct of the review and did the report justify any significant deviations from the protocol? 3. Did the review authors explain their selection of the study designs for inclusion in the review? 4. Did the review authors use a comprehensive literature search strategy? 5. Did the review authors perform study selection in duplicate? 6. Did the review authors perform data extraction in duplicate? 7. Did the review authors provide a list of excluded studies and justify the exclusions? 8. Did the review authors describe the included studies in adequate detail? 9. Did the review authors use a satisfactory technique for assessing the risk of bias (RoB) in individual studies that were included in the review? 10. Did the review authors report on the sources of funding for the studies included in the review? 11. If meta-analysis was performed, did the review authors use appropriate methods for statistical combination of results? 12. If meta-analysis was performed, did the review authors assess the potential impact of RoB in individual studies on the results of the meta-analysis or other evidence synthesis? 13. Did the review authors account for RoB in primary studies when interpreting/discussing the results of the review? 14. Did the review authors provide a satisfactory explanation for, and discussion of, any heterogeneity observed in the results of the review? 15. If they performed quantitative synthesis did the review authors carry out an adequate investigation of publication bias (small study bias) and discuss its likely impact on the results of the review? 16. Did the review authors report any potential sources of conflict of interest, including any funding they received for conducting the review

The rating of overall confidence (OC) was categorized, depending on total number flaws and weaknesses, as follows:

Critically low: More than one critical flaw with or without non-critical weaknesses: the review has more than one critical flaw and should not be relied on to provide an accurate and comprehensive summary of the available studies

Low: One critical flaw with or without non-critical weaknesses: the review has a critical flaw and may not provide an accurate and comprehensive summary of the available studies that address the question of interest

Moderate: More than one non-critical weakness: the systematic review has more than one weakness but no critical flaws. It may provide an accurate summary of the results of the available studies that were included in the review. Note: multiple non-critical weaknesses may diminish confidence in the review and it may be appropriate to move the overall appraisal down from moderate to low confidence

High: No or one non-critical weakness: the systematic review provides an accurate and comprehensive summary of the results of the available studies that address the question of interest

*Abbreviations*: *Y* Yes, criterion fulfilled, *N* No, criterion not fulfilled, *PY* Partial Yes, criterion partially fulfilled

### Summary results for exercises in chronic neck pain

The narrative analyses of the included SRs showed positive effects for all exercise types regarding pain in the short-term and when compared with non-exercise controls, and either varying or positive effects in the intermediate/long-term. For disability, all showed positive effects in the short-term compared to non-exercise controls, while compared with other exercise interventions there were no, varying, or positive effects. In the intermediate/long-term there were mainly no or varying results for pain as well as disability levels when compared to non-exercise controls as well as other exercise interventions. Our meta-analyses were based on fewer SRs (*n* = 16) but were mostly consistent with the narrative analyses. For yoga, no results concerning pain and disability in the intermediate/long-term were available.

In all, we found low- to high-quality evidence that the exercise types studied in this SR of SRs are effective for reducing pain and disability in the short-term compared to non-exercise controls, but we found conflicting results when compared to other exercises as well as in the long-term perspective (Table 4).

**Table 4:** Certainty of evidence (GRADE) for the exercise types (motor control (MCE), resistance training, traditional Chinese exercises (TCE) and yoga) compared with non-exercising and exercising control groups, for the outcomes pain and disability, at short and intermediate/long-term follow-up

**NON-EXERCISING COMPARISON GROUP**													
**Type of exercise**	**Outcome**		**Effects based on narrative analyses**^**1**^	**Effects based on meta-synthesis** **SMD (95%CI)** **(number of studies**^**2**^**)**	**Study design**^**3**^	**GRADE FACTORS**^**4**^							**Certainty of evidence**^**5**^
		**Timeframe**				**Downgrading factors**					**Upgrading factors**		
						a)	b)	c)	d)	e)	f)	g)	
**Motor Control Exercises**	**PAIN**	Short-term	positive	positive effect -1.69 (-2.73; -0.64) (2 studies)	** + + + + **	0	0	0	0	0	0	0	+ + + +
		Intermediate/long-term	varying	N/A		0	-	0	0	0	0	0	+ + +
	**DISABILITY**	Short-term	positive	positive effect -2.26 (-3.38; -1.39) (2 studies)		0	0	0	0	0	0	0	+ + + +
		Intermediate/long-term	positive	N/A		0	0	0	0	0	0	0	+ + + +
**Resistance training**	**PAIN**	Short-term	positive	positive effect -0.75 (-1.41; -0.09) (2 studies)	** + + + + **	-	0	0	0	0	0	0	+ + +
		Intermediate/long-term	positive	no effect -0.19 (-0.48; 0.09) (2 studies)		-	-	0	0	0	0	0	+ +
	**DISABILITY**	Short-term	no	no effect -0.91 (-2.22; 0.39) (2 studies)		-	0	0	0	0	0	0	+ + +
		Intermediate/long-term	no	positive effect -0.19, 95%CI -0.33 to -0.05 (2 studies)		-	-	0	0	0	0	0	+ +
**Traditional Chinese Exercises**	**PAIN**	Short-term	positive	positive effect -0.63 (-0.95; -0.32) (4 studies)	** + + + + **	-	-	0	0	0	0	0	+ +
		Intermediate/long-term	positive	positive effect -0.54 (-0.74; -0.35) (4 studies)		-	0	0	0	0	0	0	+ + +
	**DISABILITY**	Short-term	positive	positive effect -0.39 (-0.65; -0.13) (2 studies)		-	0	0	0	0	0	0	+ + +
		Intermediate/long-term	positive	positive effect -0.45 (-0.76; -0.14) (3 studies)		-	-	0	0	0	0	0	+ +
**Yoga**	**PAIN**	Short-term	positive	positive effect -1.32 (-1.84; -0.80) (2 studies)	** + + + + **	-	-	0	0	0	0	0	+ +
		Intermediate/long-term	N/A	N/A		N/A	N/A	N/A	N/A	N/A	N/A	N/A	N/A
	**DISABILITY**	Short-term	positive	positive effect -1.00 (-1.47; -0.54) (2 studies)		-	0	0	0	0	0	0	+ + +
		Intermediate/long-term	N/A	N/A		N/A	N/A	N/A	N/A	N/A	N/A	N/A	N/A
**EXERCISING COMPARISON GROUP**													
**Type of exercise**	**Outcome**		**Effects based on narrative analyses**^**1**^	**Effects based on meta-synthesis** **SMD (95%CI)** **(number of studies**^**2**^**)**	**Study design**^**3**^	**GRADE FACTORS**^**4**^							**certainty of evidence**^**5**^
						**Downgrading factors**					**Upgrading factors**		
						a)	b)	c)	d)	e)	f)	g)	
**Motor Control Exercises**	**PAIN**	Short-term	Varying	positive effect -0.25 (-0.38; -0.13) (6 studies)	** + + + + **	0	-	0	0	0	0	0	+ + +
		Intermediate/long-term	Varying	N/A		0	-	0	0	0	0	0	+ + +
	**DISABILITY**	Short-term	Varying	positive effect -0.36 (-0.52; -0.20) (4 studies)		0	-	0	0	0	0	0	+ + +
		Intermediate/long-term	Varying	N/A		0	-	0	0	0	0	0	+ + +
**Resistance training**	**PAIN**	Short-term	Varying	no effect -0.48 (-1.11; 0.15) (2 studies)	** + + + + **	-	0	0	0	0	0	0	+ + +
		Intermediate/long-term	Positive	N/A		-	0	0	0	0	0	0	+ + +
	**DISABILITY**	Short-term	No	N/A		-	0	0	0	0	0	0	+ + +
		Intermediate/long-term	No	N/A		-	0	0	0	0	0	0	+ + +
**Traditional Chinese Exercises**	**PAIN**	Short-term	Varying	no effect 0.08 (-0.09; 0.26) (3 studies)	** + + + + **	-	-	0	0	0	0	0	+ +
		Intermediate/long-term	No			-	0	0	0	0	0	0	+ + +
	**DISABILITY**	Short-term	No	no effect -0.05 (-0.26; 0.35) (2 studies)		-	0	0	0	0	0	0	+ + +
		Intermediate/long-term	No			-	0	0	0	0	0	0	+ + +
**Yoga**	**PAIN**	Short-term	Varying	N/A	** + + + + **	-	0	-	0	0	0	0	+ +
		Intermediate/long-term	N/A	N/A		N/A	N/A	N/A	N/A	N/A	N/A	N/A	N/A
	**DISABILITY**	Short-term	Varying	N/A		-	0	0	0	0	0	0	+ + +
		Intermediate/long-term	N/A	N/A		N/A	N/A	N/A	N/A	N/A	N/A	N/A	N/A

GRADE factors: a) Study limitations, b) Inconsistency, c) Indirectness, d) Imprecision, e) Publication bias, f) Moderate/large effect size, g) Dose effect

^1^Narrative analyses:

positive: all SRs/outcomes had significant positive results;

negative: all SRs/outcomes had significant negative results;

no effect: all SRs/outcomes did not show any significant results;

varying: some SRs had positive, while other SRs had negative or no significant results

N/A: not applicable, no SRs or no data available with this specific outcome

^2^SMD = standardized mean difference and the number of SRs or number of meta-analyses (outcomes) in the same SR

^3^Study design/Phase of investigation decides the starting point of the level of certainty of evidence

^4^Grade factors:

0: no reason for downgrading or upgrading;—reason for downgrading; N/A no SRs available

^5^Certainty of evidence

High (+ +  + +): We are very confident that the true effect lies close to that of the estimate of the effect

Moderate (+ + +): We are moderately confident in the effect estimate: The true effect is likely to be close to the estimate of the effect, but there is a possibility that it is substantially different

Low (+ +): Our confidence in the effect estimate is limited: The true effect may be substantially different from the estimate of the effect

Very low ( +): We have very little confidence in the effect estimate: The true effect is likely to be substantially different from the estimate of effect

### Results for various exercise types

#### MCE and pillar exercises

Eight SRs were included, and these were based on 97 studies (Tables 1 and 5). In these studies, a total of 4,566 participants were included (overlap not accounted for). Taking overlap into consideration 38 original studies were included. The SRs investigated MCE mostly using a cranio-cervical flexion hold in patients suffering from chronic neck pain [21, 26, 42–47]. Pillar exercises, which are intended to develop the ability of the spine to maintain a neutral position during load, were investigated in one high-quality SR [26]. The included SRs were published between 2010 [43] and 2023 [45]. The last updated search in the SRs was on September 30, 2022 [45]. Six SRs [21, 42, 44–47] performed a MA. The quality of the included SRs varied from low [43], to moderate [21, 44–47], to high [26, 42], and there was a very high overlap with a CCA of 21%.

**Table 5:** Results of the different exercise types compared to control interventions for pain and disability

**Motor Control Exercises (incl. Pillar exercises)**					
**Author (year)** **Study quality**	**Number of included studies (number of included studies in a meta-analysis)**	**Outcome measures** **Follow up time**	**Results pain**	**Results disability**	**Original review of authors’ conclusions**
Ferro Moura Franco, et al. (2020) [42] AMSTAR-2 High	**SR (MA)** *N* = 6 (3)	**Pain:** NPRS, VAS **Disability:** NR **Follow-up:** Short-term 6 wks -4 mo	**MCE = strength training** *Short-term* WMD = -3.16 (95% CI -13.78; 7.47) (3 studies)	**NR**	No difference between MCE and strengthening exercises. The exercises can be performed in supervised sessions with 30- to 60-min duration, 2–3 sessions/wk for a period of up to 10 wks
Garzonio, et al. (2022) [21] AMSTAR-2 Moderate	**SR (MA)** *N* = 16 (9)	**Pain:** NRS, VAS **Disability:** NR **Follow-up:** Short-term > 5 min. to ≤ 12 wks	**MCE ≥ GE (active exercises)** *Short-term* SMD = -0.31 (95% CI -0.67; 0.04) (9 studies) **MCE = Strength training** SMD = -0.10 (95% CI -0.37; 0.17) (4 studies) **MCE = Other exercises** SMD = -0.41 (95% CI -1.15; 0.32) (5 studies)	**NR**	Future studies with high methodological quality are necessary to reach firm conclusions
Hanney, et al. (2010) [43] AMSTAR-2 Low	**SR (MA)** *N* = 2 (0)	**Pain:** NR **Disability:** NDI **Follow-up:** Short-term: 6–7 wks Intermediate term: 6 mo	**NR**	**MCE = strength/endurance training/GE** *Short/intermediate term* (2 studies)	Little evidence that motor control exercises are more beneficial in either the short-term or the long-term in comparison to a more general exercise program
Martin-Gomez, et al. (2019) [44] AMSTAR-2 Moderate	**SR (MA)** *N* = 10 (10)	**Pain:** NRS, VAS **Disability:** NDI **Follow-up:** Short term 0–10 wks	**MCE = strength/endurance training** *Short term* SMD = -0.25 (95% CI – 0.50; 0.01) (5 studies) **MCE = mobilization** *Short term* *SMD* = -1.21 (95% CI -2.57; 0.15) (2 studies) **MCE > all controls** *Short term* SMD = -0.58, (95% CI -0.97; -0.20) (7 studies)	**MCE = strength/ endurance training** *Short term* SMD = -0.23 (95% CI -0.49; 0.02) (5 studies) **MCE = mobilization** *Short term* SMD = -1.44 (95% CI -3.58; 0.71) (2 studies) **MCE > all controls** *Short term* SMD = -0.44 (95% CI -0.81; -0.08) (8 studies)	Cranio-cervical flexion (MCE) compared with other treatments shows statistically significant results regarding the diminution of pain and disability in non-specific chronic neck pain GRADE very low to low certainty of effect for pain and disability
Mueller, et al. (2023) [45] AMSTAR-2 Moderate	**SR (MA)** *N* = 21 (21)	**Pain:** NRS, NPSS, VAS **Disability:** NDI **Follow-up:** Short term 2–12 wks after baseline	**MCE > control, minimal intervention** *Short term* SMD = -2.29 (95%CI -3.82; -0.75) (8 studies) **MCE = other exercise** *Short term* SMD = -0.09 (95%CI -0.61; 0.44) (12 studies)	**MCE > control, minimal intervention** *Short term* SMD = -2.42 (95%CI -3.38; -1.47) (8 studies) **MCE > other exercise** *Short term* SMD = -0.70 (95%CI -1.23; -0.17) (10 studies)	Compared to true control, effects on pain and disability were significantly larger for MCE. Higher frequencies and longer duration of MCE sessions had a significant effect on pain
Price, et al. (2020) [26] Motor Control Exercises AMSTAR-2 High	**SR (MA)** *N* = 10 (0)	**Pain:** VAS **Disability** NDI **Follow up**: Immediate ≤ 24 h Short-term > 24 h to ≤ 3 mo Intermediate term 3–12 mo	**MCE > MT** *Immediate* (1 study) **MCE > no treatment, usual care,** *Short-term* (2 studies) **MCE = general active range of movement** *Short-term* (2 studies) **MCE = other exercises and proprioceptive training** *Short-term* (4 studies) **MCE < combinations of MC, segmental exercise** *Short-term* (1 study) **MCE < Pillar exercises performed with therapist’s resistance** *Short-term* (1 study) **MCE = usual care** *Intermediate* (1 study) **MCE < pillar exercises** *Intermediate* (1 study)	**MCE = proprioceptive training** *Immediate* (1 study) **MCE > no treatment, usual care, general active range of movement, pillar exercise** *Short-term* (5 studies) **MCE = other exercises and proprioceptive training** *Short-term* (4 studies) **MCE < Pillar exercises performed with therapist’s resistance** *Short-term* (1 study) **MCE = usual care** *Intermediate* (1 study) **MCE < pillar exercises** *Intermediate* (1 study)	Based on very low-level evidence, MCE *are not* effectively reducing immediate pain Based on moderate-level evidence, MCE are not effectively reducing short-term pain or disability Based on low-level evidence, MCE are not effective in reducing intermediate-term pain or disability When MCE were used alone the effectiveness was unclear, however, benefits are maximized, when combined with other exercises
Price, et al. (2020) [26] Pillar exercises AMSTAR-2 High	**SR (MA)** *N* = 5 (0)	**Pain:** VAS (at rest, maximum, during activities) **Disability:** NDI **Follow-up** Short/intermediate term 1 wks to 3 mo	**Pillar > education** Regardless of exercise dosage *Short-term* (1 study) **Pillar < other exercises** *Short-term* (4 studies) **Pillar > education** Regardless of exercise dosage *Intermediate* (1 study) **Pillar > other exercises** *Intermediate* (1 study)	**Pillar > education** Regardless of exercise dosage *Short-term* (1 study) **Pillar < other exercises** *Short-term* (4 studies) **Pillar > education** regardless of exercise dosage *Intermediate* (1 study) **Pillar > other exercises** *Intermediate* (1 study)	Based on moderate-level evidence (GRADE) Pillar exercises are not effective in reducing short-term pain or disability compared to other exercises. Pillar exercises based on one study are effective in short-term pain and disability compared to education
Tsiringakis, et al. (2020) [46] AMSTAR-2 Moderate	**SR (MA)** *N* = 15 (10)	**Pain:** NRS, NPRS, VAS **Disability:** NDI **Follow-up:** Short-term 4–12 wks	**MCE > GE** *Short-term* Hedges´g = 0.32 (95% CI 0.04; 0.60) (9 studies)	**MCE > GE** *Short-term* Hedges´g = 0.40 (95% CI 0.12; 0.68) (10 studies)	Motor control training (with PBU) is an effective intervention for improving pain intensity and disability in patients with neck pain and is preferable to strength-endurance training of cervical muscles
Villanueva-Ruiz, et al. (2022) [47] AMSTAR-2 Moderate	**SR (MA)** *N* = 12 (11)	**Pain:** NRS, VAS **Disability:** NDI **Follow-up:** Short-term 4–12 wks Intermediate term 18–26 wks	**MCE > GE** *Short-intermediate term* SMD = -0.41 (95% CI -0.76; -0.06) (11 studies) **MCE = GE** *Intermediate* SMD = -1.30 (95% CI -3.35; 0.75) (3 studies)	**MCE > GE** *Short-intermediate term* SMD = -0.41 (95% CI -0.78; -0.04) (11 studies) **MCE = GE** *Intermediate* SMD = -1.81 (95% CI -4.29; 0.67) (3 studies)	Evidence suggests specific neck exercises are more effective than other forms of exercise, although evidence is overall of low quality
**Pilates**					
**Author (year)** Study quality	**Number of included studies (number of included studies in a meta-analysis)**	**Outcome measures** **Follow up time**	**Results pain**	**Results disability**	**Original review authors’ conclusions**
Martini (2022) [23] AMSTAR-2 High	**SR (MA)** ***N* = 5 (5)**	**Pain:** VAS **Disability:** NDI **Follow-up:** Short-term: End of intervention (6–12 weeks) Intermediate term: 6 mo	**Pilates = Other treatments/exercises** *Short-term* (5 studies) SMD = 9.29 (95% CI -25.84; 7.26) **Pilates > pharmacological treatment** *Intermediate term* (1 study) SMD = 3.11 (95% CI 2.05; 0.17)	**Pilates = Other treatments/exercises** *Short-term* (5 studies) SMD = 3.20 (95% CI -7.70; 1.30) **Pilates > pharmacological treatment** *Intermediate term* (1 study) SMD = 11.21 (95% CI 5.58; 16.74)	Based on low-certainty evidence, the Pilates method does not appear to be better than other treatments or exercises for pain and disability in the short-term for patients with neck pain
**Resistance training**					
**Author (year)** Study quality	**Number of included studies (number of included studies in a meta-analysis)**	**Outcome measures** **Follow up time**	**Results pain**	**Results disability**	**Original review authors’ conclusions**
Bertozzi, et al. (2013) [48] AMSTAR-2 Low	**SR (MA)** *N* = 9 (7)	**Pain:** VAS, NRS, Self-reported pain **Disability:** Instrument measuring the impact of chronic NP on everyday life, beyond work or leisure-time activities **Follow-up:** Short-term: < 1 mo Intermediate term: 1–6 mo Long-term: > 6 mo	**Resistance training > No intervention/education** *Short-term* Hedges’ *g* = -0.53 (95%CI -0.86; -0.20) (6 studies, 9 comparisons) *Intermediate term* Hedges’ g = -0.45 (95%CI -0.82; -0.07) (5 studies, 7 comparisons) **Resistance training = No intervention/education** *Long-term follow-up* Hedges’g = -0.04 (95%CI -0.28; 0.20) (1 study)	**Intervention = No intervention/education** *Short-term* Hedges’ g = -0.39 (95%CI -0.86; 0.07) (4 studies) *Intermediate term* (3 studies) Hedges’ g = -0.46 (95%CI -1.00; 0.08) **Intervention = No intervention/education** *Long-term follow-up* Hedges’ g = -0.14 (95%CI -0.38; 0.11) (1 study)	Consistent with other reviews, the results support the use of therapeutic exercises in the management of chronic non-specific neck pain. A significant overall effect size was found supporting therapeutic exercises for their effect on pain in both the short and intermediate terms
Cheng, et al. (2015) [49] AMSTAR-2 Crit. Low	**SR (MA)** *N* = 6 (0)	**Pain:** VAS, Pressure pain threshold, NRS, Pain Frequency **Disability:** NDI, Health-Related Quality of Life, Fear avoidance beliefs **Follow-up:** Short-term: 10 wks Long-term: 1 yr	**Resistance training > Stretching, education, and stress management** *Short-term* (2 studies) *Long-term* (1 study) **Resistance training = Control** *Long-term* (2 studies)	**Resistance training > Stretching, education, and stress management** *Long-term* (1 study)	Long-term exercise has long-term benefits for patients with nonspecific neck pain
Louw, et al. (2017) [50] AMSTAR-2 Low	**SR (MA)** *N* = 8 (2)	**Pain: intensity scale (0–9) or VAS (0–100)** **Disability:** NDI **Post-intervention:** Short-term: 10–20 wks Long-term: at 12 mo	**Resistance training > Education/stretching** *Short-term* (2 studies). NR *Long-term* SMD = -0.33 (95%CI -0.53; -0.13) (2 studies)	**Intervention = Education/stretching** NR *Long-term* SMD = -0.18 (95%CI -0.38; 0.02) (2 studies)	The overall effect shows that strengthening exercise can have a significant effect on pain reduction for up to 12 mo after the intervention is completed
Mueller, et al. (2023) [45] AMSTAR-2 Moderate	**SR (MA)** ***N* = 23 (23)**	**Pain:** NPRS (0–10), NRS or VAS (0–100) Disability: NDI, NPAD, NPNPQ, Neck disability scale (0–10) Follow-up: Short-term: 4–12 wks (1 study 24 wks)	**Resistance training > real control/minimal intervention** *Short-term* SMD = -1.27 (95%CI -2.26; -0.28) (5 studies) **Resistance training = other exercise** *Short-term* SMD = -0.31 (95%CI -1.05; 0.44) (16 studies)	**Resistance training > real control/minimal intervention** *Short-term* SMD = -1.76 (95%CI -3.16; -0.37) (5 studies) **Resistance training = real control/minimal intervention** *Short-term* SMD = 0.40 (95%CI -0.18; 0.98) (14 studies)	Resistance and motor control exercises were effective for reducing neck pain (very low– to moderate-certainty evidence). Higher frequencies and longer duration of sessions had a significant effect on pain for motor control exercise
Seo et al. (2020) [52] AMSTAR-2 Crit low	**SR (MA)** *N* = 4 (0)	**Pain:** VAS, Self-rated pain intensity **Disability:** NDI **Follow-up:** Short-term: Post-intervention	**Resistance training > Control (NR)** *Short-term* (2 studies)	**Intervention = Control (NR)** *Short-term* (1 study)	Scapular stabilization exercises could be considered an effective intervention for patients with nonspecific neck pain. Scapular stabilization may improve neck pain and function, but the evidence of this in the reviewed articles was insufficient
Ouellet, et al. (2021) [51] AMSTAR-2 High	**SR (MA)** *N* = 4 (4)	**Pain:** VAS **Disability:** NDI **Follow-up:** **Short term:** 6–13 wks **Intermediate:** 4–9 mo	*Short-term* **Region-specific exercises = General exercises** MD = -0.86 (95% CI -2.20; 0.48) (4 studies) **Region-specific exercises = Aerobic exercises** MD = -1.16 (95%CI -3.83; 1.52) (2 studies) *Intermediate* **Region-specific exercises > Tai chi, Dantian Qigong exercise** (2 trials) **Region-specific exercises > Aerobic exercise** (1 study) **Region-specific exercises > Aerobic exercise** (1 study)	*Short term* **Region-specific exercises = Tai chi, Strength training + proprioceptive exercise group, Dantian Qigong exercises** (2 studies) *Intermediate* **Region-specific exercises = Tai chi, Strength training + proprioceptive exercise group, Dantian qigong exercises** (2 studies)	The difference in treatment effect remains uncertain between region-specific and general exercises. Based on very low to low-quality evidence, there appear to be no differences between both types of exercise approaches for pain reduction or disability for adults with spinal disorders
Price, et al. (2020) [26] Resistance Exercises AMSTAR-2 High	**SR (MA)** *N* = 2 (0)	**Pain:** VAS (at rest, maximum, during activities) **Follow-up** Short/intermediate term 1 wks to 3 mo **Follow-ups** Short-term Intermediate	**ULRE > No treatment** *Short-term* (1 study) **ULRE > education/stress reduction***Short-term* (1 study; 3 outcomes) **ULRE < education/stress reduction***Intermediate* (1 study) **ULRE > Other exercise** *Short-term* (1 study) **ULRE = Other exercise** *Short-term* (1 study) **ULRE > Body awareness training***Short-term* (1 study; 2 outcomes) **ULRE = Body awareness training***Short-term* (1 study) *Intermediate* (1 study; 2 outcomes)	**NR**	Based on low-level evidence (GRADE) ULRE are effective in reducing pain in the short term but not in the intermediate or long term
Yang, et al. (2022) [53] AMSTAR-2 Low	**SR (MA)** *N* = 18 (18)	**Pain:** VAS **Disability:** NDI	**Isometric exercises > controls** *Short-term* WMD = -0.81 (95%CI -0.88; -0.73) (16 studies)	**Isometric exercises < controls** WMD = -5.55 (95%CI -4.57; -6.53) (10 studies)	Isometric training can reduce the degree of neck pain and improve neck dysfunction. An intervention frequency of more than 20 times had more significant improvement effects on the degree of neck pain, compared to an intervention frequency of less than 20 times. In addition, the effect of isometric training on VAS and NDI indices with an intervention period of more than 8 weeks was more significant compared to a training period of less than 8 weeks
**Traditional Chinese Exercises**					
**Author (year)** Study quality	**Number of included studies (number of included studies in a meta-analysis)**	**Outcome measures** **Follow up time**	**Results pain**	**Results disability**	**Original review authors' conclusions**
Bai, et al. (2015) [54] AMSTAR-2 Low	**SR (MA)** *N* = 2 (2)	**Pain** VAS **Follow-up:** Short-term: After treatment (3 mo.) Intermediate term: 6 mo. after study-start	**TCE > Waiting list control** *Short-term* SMD: -1.17 (95% CI: -2.44; 0.1) (2 studies) *Intermediate-term* **TCE > Waiting list control** SMD: -1.00 (95% CI: -1.94, -0.06) (2 studies)	**NR**	Significant difference for internal Qigon for chronic neck pain compared to waiting list controls at 3 and 6 months
Ferro Moura Franco, et al. (2020) [42] AMSTAR-2 High	**SR (MA)** *N* = 4 (3)	**Pain** VAS **Follow-up** Short-term: post-intervention 3 mo	**TCMBE < Exercise therapy** *short-term* MD = 6.46 (95%CI 0.75; 12.17) (3 studies)	**NR**	Combined exercise was better than meditative therapies (Tai Chi and Qigong). Focused and intense exercises for a short period of time can be prescribed for patients with nociceptive pain predominance
Girard, et al. (2019) [55] AMSTAR-2 Critically low	**SR (MA)** *N* = 3 (0)	**Pain:** VAS **Disability:** NDI, NPDS **Follow-up** 3 mo and at 6 mo	*Short term* **Qigong = Waiting list controls** (1 study) **Qigong = Exercise therapy** (2 studies) *Intermediate term* **Qigong > Waiting list controls** (1 study) **Qigong = Exercise therapy** (1 study)	*Short term* **Qigong = Waiting list controls** (1 study) **Qigong = Exercise therapy** (2 studies) *Intermediate term* **Qigong > Waiting list controls** (1 study) **Qigong = Exercise therapy** (1 study)	The findings of this systematic review indicate that qigong might have a beneficial effect in some individuals with neck pain, although not necessarily more effective than therapeutic exercise
Gross, et al. (2016) [32] AMSTAR-2 Moderate	**SR (MA)** *N* = 2 (2)	**Pain:** VAS **Disability**: NPDI and NDI **Follow-up:** Short-term 12 wks Intermediate 24 wks	**Qigong > Waiting list controls** *Short-term* Pooled MD = -13.28 (95%CI -20.98; -5.58) (2 studies) **Qigong > Waiting list controls** *Intermediate* Pooled MD = -7.82 (95%CI -14.57; -1.07) (2 studies)	**Qigong > Waiting list controls** *Short-term* Pooled MD = -0.36 (95%CI -0.68; -0.03) (2 studies) **Qigong = Waiting list controls** *Intermediate* Pooled MD = -0.28 (95%CI -0.68; 0.11) (2 studies)	**Moderate quality evidence**: Two trials show that Qigong exercises (Dantian Qigong) may improve pain and function slightly when compared with waiting list controls at short-term follow-up
Kong et al. (2022) [61] AMSTAR-2 Low	**SR (MA)** **N = 4 (3)**	**Pain:** VAS **Disability:** NDI **Follow-up:** Short term 3 mo Intermediate term	**TCE > Waiting list controls** *Short term* (3 studies)^a^ SMD = 0.81 (95%CI -1.13; -0.50) **TCE = Other exercises** *Short term* (3 studies)^a^ SMD = -0.07 (95%CI -0.33; 0.18) **TCE > other treatments** *Intermediate term* (3 studies) ^a^ SMD = -0.69 (95%CI -0.39; -0.99)	**TCE > other treatments** *Short term* (2 studies) SMD = 0.74 (95%CI 0.40; 1.08)	The evidence supporting the effects of TCEs alone in improving pain and disability in patients with neck pain was limited due to the small sample size. The follow-up effects of TCEs were still insufficient
Xie, et al. (2021) [56] AMSTAR-2 Low	**SR (MA)** N = 5 (5)	**Pain:** VAS **Disability:** NDI, NPDS **Follow-up:** Short term 3 mo	**TCMBE = Exercise therapy** *Short-term* MD = 1.88 (95%CI -7.70; 11.46) (5 studies)	**TCMBE = Exercise therapy** *Short-term* NDI MD = 0.15 (95%CI -6.37; 6.66) (3 studies) *Short-term* NPDS MD = 1.31 (95%CI -4.10; 6.71) (2 studies)	Based on the meta-analysis, there is insufficient evidence to support the clinical use of TCMBE in improving pain intensity and enhancing functional mobility in individuals with neck pain
Yuan, et al. (2015) [57] AMSTAR-2 Moderate	**SR (MA)** N = 3 (2)	**Pain:** VAS **Disability:** NDI, NPDS **Follow-up** Short term 3 mo Intermediate 3–12 mo	**Qigong > Waiting list controls** *Short-term* WMD = -15.27 (95%CI -22.49; -8.05) (2 studies) **Qigong > Waiting list controls** *Intermediate term* WMD = -10.18 (95%CI -16.63; -3.73) (2 studies) **Qigong = Exercise therapy** *Short term* WMD = 1.88 (95%CI -5.77; 9.54) (2 studies) **Qigong = Exercise therapy** *Intermediate term* WMD = -1.00 (95%CI -6.21; 8.21) (2 studies)	**Qigong > Waiting list controls** *Short-term* WMD = -7.67 (95%CI -12.45; -2.88) (2 studies) **Qigong = Waiting list controls** (2 studies) *Intermediate term* WMD = 0.43 (95%CI -4.43; 5.28) (2 studies) **Qigong = Exercise therapy** *Short term* WMD = 1.29 (95%CI -4.32; 6.91) (2 studies) **Qigong = Exercise therapy** *Intermediate term* WMD = 0.02 (95%CI -5.25; 5.28) (2 studies)	Two small studies showed that qigong may be superior compared with waiting list controls for chronic neck pain sufferers (moderate evidence), but no differences were found compared with other exercises
de Zoete, et al. (2020) [58] AMSTAR-2 Critically Low	**SR (MA)** **Qigong** *N* = 2 (0) **Tai-Chi** *N* = 1 (0)	**Qigong** **Pain:** NR **Disability:** NR **Follow-up:** Up to 6 mo **Tai-Chi** **Pain:** NR **Disability:** NR **Follow-up:** 6 mo post-intervention (1 study)	**Qigong = Waiting list controls** (1 study) **Qigong = Neck-specific exercise** (2 studies) **Tai-Chi > Waiting list controls** (1 study) **Tai-Chi > Neck-specific exercise** (1 study)	**Qigong NR** **Tai Chi NR**	No specific conclusions were drawn concerning TCE, however, the authors concluded that all their included studies showed positive effects on neck pain intensity and disability and that general physical exercise showed equal or superior improvements in neck pain intensity and neck disability compared to usual care alternatives
**Yoga**					
**Author (year)** Study quality	**Number of included studies (number of included studies in a meta-analysis)**	**Outcome measures** **Follow up time**	**Results pain**	**Results disability**	**Original review authors' conclusions**
Cramer, et al., (2017) [59] AMSTAR-2 High	**SR (MA)** *N* = 3 (3)	**Pain:** NRS, VAS **Disability:** NDI **Follow-up:** Short-term: NR	**YOGA > Usual care** *Short-term* SMD = -1.28 (95%CI -1.81; -0.75) (3 studies)	**YOGA > Usual care** *Short-term* SMD = -0.97 (95%CI -1.44; -0.50) (3 studies)	Yoga has short-term effects on chronic neck pain, its related disability, quality of life, and mood suggesting that yoga might be a good treatment option Meta-analysis of these trials found robust evidence for large short-term effects of (Iyengar) yoga on neck pain intensity, pain-related disability in patients with chronic non-specific neck pain
Kim, et al. (2016) [60] AMSTAR-2 Critically low	**SR (MA)** *N* = 3 (0)	**Pain:** NRS, VAS **Disability:** NDI **Follow-up:** Short term 6 wks	**YOGA > Home Exercise** *Short-term* (3 studies)	**YOGA > Home Exercise** *Short-term* (3 studies)	Significant decreases in chronic neck pain intensity and functional disability for the yoga group were found in all of the trials. These findings support the practice of yoga as an evidence-based treatment for chronic neck pain. Evidence from the three RCTs shows that yoga may be beneficial for chronic neck pain
Li, et al. (2019) [22] AMSTAR-2 Low	**SR (MA)** *N* = 10 (10)	**Pain:** CPGS NRS SF-MPQ VAS **Disability:** NDI NDS NPDS NPQ **Follow-up:** *Short-term:* post-intervention—6 wks *Intermediate:* 6 mo., *Long-term:* 12 mo	**YOGA > Other therapies (e.g., exercise, Pilates, usual care)** *Short-term* (10 studies) SMD = -1.13 (95%CI -1.60; -0.66) *Subgroup analyses* **YOGA = PILATES** *Short-term* SMD = -0.18 (95%CI -0.76; 0.39) (3 studies) **YOGA > EXERCISE** *Short-term* SMD = -1.26 (95%CI -1.83; -0.68) (9 studies) **YOGA = CAM** *Short-term* SMD = -2.40 (95%CI -5.26; 0.46) (2 studies)	**YOGA > Other therapies (e.g., exercise, Pilates, usual care)** *Short-term* SMD = -0.92 (95%CI -1.38; -0.47) (8 studies) *Subgroup analyses* **YOGA = PILATES** *Short-term* SMD = -0.27 (95%CI -0.88; 0.35) (3 studies) **YOGA > EXERCISE** *Short-term* SMD = -0.97 (95%CI -1.55; -0.38) (7 studies) **YOGA = CAM** *Short-term* SMD = -2.31 (95%CI -5.35; 0.73) (2 studies) *Long-term* NR	It was difficult to make a comprehensive summary of all the evidence due to the different sessions and duration of the yoga interventions, and the different outcome measurement tools in the study, we draw a very cautious conclusion that yoga can relieve neck pain intensity, improve pain-related function disability, increase CROM. This suggests that yoga might be an important alternative in the treatment of CNNP
De Zoete, et al. (2020) [58] AMSTAR-2 Critically low	**SR (MA)** *N* = 3 (0)	**Pain:** 100 mm VAS **Disability:** NR **Follow-up:** Short-term: Post-intervention	**Yoga > self-care** (2 studies) One study reported only within-group analyses	**Yoga > self-care** (2 studies) One study reported only within-group analyses	No specific conclusions were made about yoga, but the authors concluded that general physical exercises may be more effective than interventions lacking such exercises (i.e. only neck-specific exercises)

*Abbreviations*: *CCF* Cranio-cervical flexion, *GE* General Exercise, *MA* Meta-Analysis, *MCE* Motor Control Exercises, *mo* months, *MT* Manual Therapy, *N* Number of studies included, *n* number of subjects included, *NR* Not reported, *NRS* Numeric rating scale (0–10), *NDI* Neck Disability Index (0–100), *NPSS* Neck Pain Severity Score (0–100), *SMD* Standardized Mean Difference, *SR* Systematic review, *VAS* Visual Analogue Scale (0–100), *PBU* Pressure biofeedback, *WMD* Weighted Mean Difference, *wks* weeks, *MD* Mean Difference, *NDI* Neck Disability Index, *SMD* Standard Mean Difference, *ULRE* Upper limb resistance exercises, *CE* Core Exercises, *EPA* Electrophysical agents, *ES* Effect Size, *MI* Minimal intervention; *MvCE* Movement Control Exercises, *MCID* Minimal clinical important difference, *NDI* Neck disability index (0–50), *NPDS* Neck pain and disability scale (0–100), *NA* Not applicable, *TCMBE* Traditional Chinese Mind and Body Exercise, *CAM* Complementary and alternative medicine, *CG* Control group (overall effect), *CNNP* Chronic Non-specific Neck Pain, *CPGS* Chronic Pain Grade Scale (Pain and disability), *NDS* Neck Disability Score, *NPDS* Neck Pain and Disability Scale, *NPQ* Northwick Park Questionnaire, *RCT* Randomized controlled trial, *SF-MPQ* Short-Form McGill Pain Questionnaire, *UC* Usual care

^a^recalculated from the data presented in the paper

Seven SRs reported results on the effect of MCE on pain compared to various control treatments, including general exercises [21, 26, 45–47], strength and endurance exercises [21, 26, 42, 44, 45, 47, 53], manual therapy [26, 44], and minimal interventions such as usual care or education [26, 45, 53]. Most SRs investigated MCE in the short/intermediate perspective, while only one SR investigated the effect of the MCE in the long-term [47]. When MCE was compared to manual therapy, one high-quality SR reported that MCE was more effective in the short-term [26] while another SR with moderate quality reported no difference between MCE and manual therapy in the short/intermediate term [44]. One SR with high quality reported that MCE was more effective when compared with usual care in the short/intermediate term [26] and one SR with moderate quality showed positive results when compared to a true comparison group/minimal intervention [45]. Combining the two SRs [44, 45] that provided aggregated data using a non-exercising comparison group, we found significant positive effects for MCE on pain-intensity in the short-term (SMD = -1.69, 95%CI -2.73 to -0.64; I^2^ = 5%; Additional file 5). There was an inconsistency regarding the reported effect on pain for MCE compared to other exercise interventions, where four of the SRs with moderate to high quality reported positive effects in the short/intermediate-term [21, 44, 46, 47], while three SRs of medium and high quality [21, 44, 45] reported no results. However, combining the six SRs [21, 42, 44–47, 53] that provided aggregated data comparing MCE with other exercise interventions into a meta-analysis we found significant positive effects on pain-intensity in the short-term (SMD = -0.25, 95%CI -0.38 to -0.13; I^2^ = 0%; Additional file 5). There were no positive intermediate/long-term effects reported when comparing MCE to other exercises in one SR with moderate quality [47]. For Pillar exercises, one high-quality SR reported no effect on pain in the short/intermediate term compared with other exercise treatments, but a positive effect compared with education [26].

Six SRs reported results on disability, and of these three SRs compared MCE with a non-exercising control group in the short-term. One moderate-quality SR did not find significant results [44], while two moderate-quality SRs found positive results [26, 45]. The meta-analysis based on the two SRs that provided data [44, 45] showed significant positive effects for MCE (SMD = -2.26, 95%CI -3.13 to -1.39; I^2^ = 0%; Additional file 5). Four moderate-quality SRs [44–47] reported a positive effect of MCE compared to other exercises, while one low-quality SR [43] and one high-quality SR [26] reported no positive short-term effects. Our meta-analysis based on four SRs [44, 45, 47, 62] showed short-term positive effects of MCE compared to other exercises (SMD = -0.36, 95%CI -0.52 to -0.20; I^2^ = 0%; Additional file 5). In the intermediate-term, one moderate-quality SR reported no difference between MCE and other exercises [47], while MCE was found significantly inferior to Pillar exercises in a high-quality SR [26]. No effect was reported comparing MCE with manual therapy in the immediate term [44]. Regarding the effect of Pillar exercises, one high-quality SR showed that Pillar exercises had no positive effect compared with other exercises in the short/intermediate term, while a positive effect was reported for Pillar exercises compared to education in the short/intermediate term [26].

The GRADE analyses (Table 4) showed that there is a high certainty of evidence that there are positive effects of MCE but not of Pillar exercises on pain and disability compared to non-exercise controls in the short-term. Compared to other exercise types, there are positive results concerning the effect of MCE but not for Pillar exercises on pain and disability in the short-term. In the intermediate/long-term, there is a high certainty of evidence that MCE is more effective than non-exercise controls concerning disability, but not compared to exercise controls. Moreover, we found varying results if MCE compared to non-exercise in the intermediate/long-term as well as other exercise interventions for pain. Downgrading was mainly based on the inconsistency of the results.

#### Pilates

One high-quality SR (MA), based on 5 original studies was included [23]. In the study, a total of 224 participants with chronic neck pain were included. The included SR was published in 2022, and the search was done up until October 2021. The SR investigated Pilates interventions compared with other exercises such as stabilizing exercises, stretching, or strength training or in one of the studies with pharmacological intervention. The SR reported, based on their MA, a low certainty evidence, that the results for pain are not more positive than other exercises/treatments in the short term (SMD = 9.29, 95% CI -25.84; 7.26). The same refers to disability in the short term (SMD 3.20, 95% CI -7.70: 1.30). One of the original studies investigated Pilates in the intermediate term and reported that there is a moderate certainty evidence that Pilates is more effective than a pharmacological intervention for pain (SMD = 3.11, 95%CI 2.05; 0.17) and for disability (SMD = 11.21, 95%CI 5.58; 16.74).

#### Resistance training

Eight SRs were included, and these were based on a total of 74 studies (Tables 1 and 5). These studies included a total of 8,380 participants (overlap not accounted for) and investigated some form of isometric or dynamic resistance exercises in patients suffering from chronic neck pain [26, 45, 48–53]. Taking overlap into consideration 65 original studies were included. The included SRs were published between 2013 [48] and 2023 [45], and the last updated search in the SRs was performed in September 2022 [45]. Six out of the eight SRs performed an MA [26, 45, 48, 50, 51, 53]. The quality of the included SRs varied from critically low [49, 52], and low [48, 50, 53], to moderate [45] and high quality [26, 51]. There was nearly no overlap for resistance training (CCA = 2%). There was a large range in dosage, e.g., the number of treatment sessions, duration, number of sets and reps, and intensity. In most studies, external resistance such as dumbbells, resistance bands, or body weight were used for training specific neck and shoulder muscles. Six of the SRs included a comparison to a non-exercise control such as no treatment, education, or stretching [26, 45, 48–50, 52], and three included a comparison to another exercise-based control such as Thai Chi, aerobics, or general exercises [45, 50, 51].

Concerning pain, all six SRs that compared the effect of resistance training against a non-exercise control reported a positive effect at the short-term follow-up [26, 45, 48–50, 52]. Two SRs of moderate respective low quality [45, 48] provided data for a meta-analysis and our results showed significant short-term effects on pain-intensity in favour of resistance exercises compared to non-exercising controls (SMD = -0.75, 95%CI -1.41 to -0.09; I^2^ = 48%; Additional file 5). A low-quality SR [48] also reported positive effects in the intermediate term, but this was not confirmed by a high-quality SR [26]. Compared to a non-exercise control group, three SRs reported on long-term effects on pain, with one low-quality SR reporting positive effects of resistance training [50], one low-quality SR reporting no difference [48], and one critically low-quality SR reporting contradicting results [49]. The results were narratively found to be varying, and our meta-analysis based on two of the included SRs [48, 63] showed no positive results on pain in the intermediate/long-term for resistance training compared with non-exercise controls (SMD = -0.19, 95%CI -0.48 to 0.09; I^2^ = 70%; Additional file 5).

When narratively comparing resistance training to other exercise-based controls such as Thai Chi, aerobics, and general exercises, varying results were found in three SRs [26, 45, 51]. Our meta-analysis based on two SRs of moderate and high quality [45, 51] showed, however, no significant short-term effects for resistance exercises when compared to exercising controls SMD = -0.48 95%CI -1.11 to 0.15; I^2^ = 0%; Additional file 5). One high-quality SR reported positive effects in the intermediate term [51].

Concerning disability, two SRs with critically low and low quality that compared the effect of resistance training to non-exercise controls reported no effects at the short-term follow-up [48, 52], while one SR with moderate quality showed positive effects [45]. Our meta-analysis on two of these SRs [45, 48], showed no significant short-term effects on disability for resistance exercises when compared to non-exercising controls SMD = -0.91 95%CI -2.22 to 0.39; I^2^ = 70%; Additional file 5). Of the three SRs reporting intermediate/long-term effects, two SRs of low and critically low quality reported positive effects of resistance training [49, 50], and one low-quality SR reported no positive effect, all compared to non-exercising control groups [48]. Moreover, one high-quality SR compared a resistance intervention to other exercise-based controls and reported no positive effects in the intermediate-term follow-up [51]. Our meta-analysis based on two studies [48, 50] on the effects of resistance training in the intermediate/long-term on disability showed positive results when compared to non-exercise controls (SMD = -0.19, 95%CI -0.33 to -0.05; I^2^ = 0%; Additional file 5).

One low-quality SR (MA) [53] concluded that long-term isometric resistance exercises were effective for lowering both pain-intensity, but included mixed control groups, and did not report if the outcomes regarded short- or long-time outcomes and was therefore not included in our narrative synthesis or meta-analyses [53].

The GRADE analyses showed (Table 4) that there is moderate certainty of evidence that, compared to non-exercise controls, resistance training has a positive effect on pain in the short-term and that there is low certainty of evidence for a positive effect on disability in the intermediate/long-term. However, compared to exercise controls in the short- and intermediate/long-term, there is evidence of moderate certainty that resistance training is not better. The certainty of evidence was downgraded due to low study quality and inconsistent results.

#### TCE

Eight SRs were included, and these were based on 26 studies (Tables 1 and 5). The eight SRs included a total of 2,905 participants (overlap not accounted for) and investigated the effect of TCE (Qigong and Tai Chi) in patients suffering from chronic neck pain [32, 42, 54–58, 61]. Taking overlap into consideration 7 original studies were included. The included SRs were published between 2015 [54, 57] and 2022 [61], and the last updated search in the SRs was performed in January 2022 [61]. There was a very high overlap for TCE with a CCA of 41%. The quality of the included SRs varied from critically low [55, 58], low [54, 56, 61], moderate [32, 57] to high quality [42]. Both Qigong and Tai Chi interventions were included in the SRs. The type of Qigong varied and included Dantian, Neiyanggong, and Biyun Medical Qigong, but also included neck- and shoulder exercises and in addition moving and breathing exercises. Three SRs included Tai Chi based on the Yang style, all with different combinations of body posture, movement, breathing, meditation, relaxation, and self-massage [42, 56, 58]. Qigong was compared with other exercise types including softball and TheraBand exercises, strength and endurance training, flexibility/mobility exercises, proprioceptive exercises, neck-specific exercises, and cervical manipulation [55, 57, 58, 61]. However, most studies compared TCE to waiting list controls that received no or only minimal intervention [32, 54, 55, 57, 58, 61].

Six of the included SRs reported results on pain with a focus on Qigong and Tai Chi compared with non-exercising controls [32, 54–58]. TCE showed positive effects compared with wait-list controls in the short-term in three SRs, one (TCE) with low quality [54] and two (Qigong) with moderate quality [32, 57]. One SR (Qigong) with critically low study quality found no difference with the non-exercising control [55]. One SR, also with critically low study quality [58] showed varying results, in which Qigong was found to be no better than waiting list control, while Tai Chi showed positive results. Our meta-analysis of the available data in four of the included SRs [20, 54, 57, 61] showed significant positive short-term effects of TCE on pain compared with non-exercising controls (SMD = -0.63, 95%CI -0.95 to -0.32; I^2^ = 30%; Additional file 5). TCE was also found to be superior to non-exercising controls in the intermediate term in four SRs [32, 54, 57, 61] with SMD = -0.54, 95%CI -0.74 to -0.35; I^2^ = 3%; Additional file 5. Five SRs reported varying results on TCE compared with different exercise controls [42, 55–58]. Thai Chi showed positive effects compared with neck-specific exercises in the short-term in one SR with critically low study quality [58]. Compared with other exercise interventions, TCE did not show any positive effects in four SRs with critically low to moderate quality [55–58], while one SR with high study quality [42] reported that other exercise interventions were superior compared with TCE. Our meta-analysis based on data from 4 of the included SRs found non-significant results (SMD = 0.08, 95%CI -0.09 to 0.26; I^2^ = 19%; Additional file 5) [42, 56, 57, 61].

Five of the included SRs reported results on disability [32, 55–57, 61]. Qigong was found to be superior to non-exercising controls in the short-term in two SRs with moderate study quality [32, 57] while one SR [55] with low study quality found Qigong to be no better than waiting list controls. Based on data available from 2 SRs [32, 57], our meta-analysis showed significant positive short-term results on disability for TCE compared to non-exercising controls (SMD = -0.39, 95%CI -0.65 to -0.13; I^2^ = 0%; Additional file 5). In the intermediate term, Qigong was found to be superior to non-exercise controls in the intermediate term in one SR [55] while two SRs found Qigong to be no better than waiting list controls [32, 57] and our meta-analysis based on these SRs showed significant short-term effects on disability for TCE compared to non-exercise controls (SMD = -0.45, 95%CI -0.76 to -0.14, I^2^ = 52%; Additional file 5). Three SRs reported results on disability where TCE was compared with various exercising controls [55–57]. TCE was no better than exercise therapy in the short-term in one SR with low study quality [56] while Qigong was no better than exercise therapy in the short- as well as in the intermediate term [55, 57]. Based on data from two SRs with critically low to moderate study quality [55, 57] our meta-analysis showed no effects of short-term effects of TCE compared to exercise controls (SMD = 0.05, 95%CI -0.26 to 0.35, I^2^ = 0%; Additional file 5).

The GRADE analyses showed (Table 4) that there is low to moderate certainty of evidence that TCE with a focus on Qigong and/or Tai Chi has a positive effect on pain in short- and intermediate/long-term and on disability in the short-term compared to non-exercise controls. The level of evidence was downgraded due to low study quality and inconsistency. With low certainty of evidence, we found positive results for intermediate/long-term effects on disability compared to non-exercise controls. There is a moderate certainty of evidence that TCE is not effective compared to exercising controls on pain in the intermediate/long term and for disability in both the short- and long-term. The level of evidence was downgraded due to low study quality. Low certainty of evidence was found for varying results on pain when compared to exercising controls.

#### Yoga

Four SRs were included, and these were based on 10 studies (Tables 1 and 5). The SRs included a total of 1,246 participants (overlap not accounted for) and investigated some form of yoga in patients suffering from chronic neck pain [22, 58–60]. Taking overlap into consideration 10 original studies were included. The SRs were published between 2016 [60] and 2020 [58], and the last updated search in the SRs was in 2018 [22, 58]. Two of the four SRs performed an MA [22, 59]. The quality of the included SRs varied from critically low quality [58, 60], to low quality [22], to high quality [59]. There was a very high overlap with a CCA of 30%. No SR investigated the effect of yoga from a long-term perspective.

The SRs included different yoga styles, and these could include combinations of physical postures, breathing, and meditation with the aim of promoting well-being. The most-studied yoga style was Iyengar yoga (a Hata yoga, which implies a more physical-based style) which uses protocols that focus on postures (asanas) that lengthen and strengthen muscles in the neck and shoulders to improve stability, flexibility, alignment, and mobility in muscles, joints, and tendons combined with breathing regulation (pranayama) and relaxation (dyana). Some studies included Kriya and Kundalini yoga, in which one relies less on the asanas and more on energy management, meditation, and breathing techniques [59], but also lesser-known programs like the yogic mind sound resonance technique, which relies on relaxation techniques practiced in supine or sitting positions aiming to increase will power, concentration, and deep relaxation [64]. The yoga interventions were heterogeneous not only in style, but also in the length, frequency, and intensity of the sessions. The interventions were given for a period of between 10 days and 3 months and lasted between 20 min per day to 90 min a week. The control interventions were treatment such as physical therapy [58, 59], exercise [22, 60], or other active non-pharmacological control interventions, Pilates exercises, usual care, self-care information, and supine rest [58–60].

The narrative synthesis on pain intensity in the included SRs showed positive short-term post-intervention effects for yoga compared with no or only minimal intervention, while our meta-analysis based on two SRs with low respective high quality [22, 59] showed positive results (SMD = -1.32, 95%CI -1.84 to -0.80; I^2^ = 0%; Additional file 5) but there were varying results compared to general exercises [22, 58–60]. Narratively, two SRs with high and low quality [22, 59] showed positive effects. Regarding disability, there were short-term positive effects for yoga compared to no or only minimal intervention in our meta-analysis based on two SRs [22, 59] with low respective high quality (SMD = -1.00, 95%CI -1.47 to -0.54; I^2^ = 0%; Additional file 5), but not compared to general exercises [22, 58–60].

The GRADE analyses showed (Table 4) that there is a low certainty of evidence for positive effects in the short-term of yoga regarding pain and a moderate certainty of evidence for positive effects in the short-term of yoga regarding disability when compared to non-exercise controls. Compared to exercising controls, there was no effect with low to moderate certainty of evidence for pain and disability, respectively. The level of evidence was downgraded due to poor study quality and conflicting results. Long-term effects could not be analyzed due to a lack of studies.

## Discussion

This SR of SRs summarized the literature on various exercise types used in treating chronic neck pain. Our results show a low to high certainty of evidence that the exercise types studied demonstrate positive effects when compared to non-exercise controls for pain levels in the short-term. For disability, all showed positive effects in the short-term compared to non-exercise controls except resistance training. Compared to other exercise interventions, MCE showed positive results for pain and disability levels, while the other exercise types showed varying, or no results. In the long-term, there were mainly no or varying results when compared to non-exercise controls as well as other exercise interventions. Only one SR investigating Pilates was found in our database search and reported, based on low-certainty evidence, that Pilates exercises are not better than other exercises in the short-term to reduce pain and disability, thus aligning with our study findings of the other exercises [23]. Our results are based on 25 SRs (including a total of 125 original studies) with varying risk of bias, including five SRs with high quality, seven SRs with moderate quality, eight SRs with low quality, and five SRs with critically low quality.

Our results partly concur with the results from two SRs on the effect of exercise on chronic neck pain [58, 65]. De Zoete et al. (2020) reported on the effectiveness of general physical exercise (individualized physical exercise, yoga and Pilates, and Tai Chi and Qigong) on pain and disability in chronic neck pain and showed that these exercises have a positive effect compared to usual care interventions [58]. Furthermore, in a network meta-analysis including 40 original trials, the authors found low-quality evidence that exercises such as MCE, yoga, and TCE are equally effective in reducing pain and disability [65]. Mueller et al. (2023) showed with very low to moderate certainty evidence that the effects of MCE and resistance exercises increase with increased frequencies and longer duration of sessions [45]. On the other hand, previous studies have shown contradicting results [26]. Additionally, an updated Cochrane review on exercises for mechanical neck disorders further concluded that exercises for neck pain are safe with only minor adverse effects, but no high-quality evidence exists for the effectiveness of these exercises [20]. Even so, exercises, often MCEs and strength/endurance exercises, are recommended and used in the treatment of patients with chronic neck pain, and often together with early advice and education as recommended [18]. The challenge thus remains for the clinician to decide what type of exercise to use and with what dosage.

In our literature search, we found several SRs reporting on the effect of MCE, and all but one was published between 2020 and 2022 thus indicating that there has been a recent research focus on this exercise type. The results from our meta-analysis show that MCE had a positive effect compared to non-exercise controls as well as exercise controls for pain and disability levels in the short-term. MCE are performed as specific exercises, and under low load affecting the postural stability, thus might be preferred in the short-term before introducing more loaded exercise types [24, 25]. When summarizing the literature, MCE seemingly comprise different methodologies. One approach – craniocervical-flexion hold – is a static approach that was investigated in most of the included SRs, often using a biofeedback device to control the hold of the neck during the exercise [21, 26, 42, 44, 46, 47]. Other exercises used a more functional approach (Pillar exercises, segmental exercises) where the neck was challenged by loaded exercises via the arms, either via resistance by pulleys or by manual resistance by the therapist [26]. A challenge in summarizing the effect of MCE was that in several studies various types of MCE were compared to each other, which could result in a lack of between-group differences.

Summarizing the SRs on resistance training, we found evidence of moderate certainty that resistance training is effective for lowering levels of pain compared to no/minimal intervention in the short-term. These positive short-term effects for pain, when compared to a non-exercising control group, are in contrast to our results from the SR of SRs on the effects of resistance training for low back pain [27], which might indicate that the treatment mechanisms differ for neck compared to back pain. We also concluded that there is moderate certainty evidence that there are no positive effects of resistance training when compared to other types of exercises and inconclusive results were found for the long-term effects. Concerning disability, we found varying results with low certainty of evidence on the effects of resistance training compared to a no/minimal intervention control group in the intermediate/long-term, and there was moderate certainty of evidence for no effects compared to other types of exercises in the short-term. In the present SR of SRs, the resistance training was very heterogeneous, which makes it impossible to give clinical recommendations on the type or dosage. Future studies should investigate different dosages and incorporate progressive loading principles identified in previous research [66].

For TCE there was low- to moderate certainty of evidence that Qigong and/or Tai Chi have positive short- and long-term effects on pain and short-term effects on disability compared to waitlist controls. However, compared to exercise controls, there was low- to moderate certainty of evidence for varying/no effects of TCE on pain and disability. The types of TCE varied largely between the different studies as well as the number of treatment sessions, duration of treatment sessions, and duration of the training period. One interesting finding in our current SR of SRs is that for chronic neck pain, we found an extremely high overlap and more SRs on TCE (seven) than there are RCTs on TCE (five). The more than 20-fold increase in the number of SRs during the last 20 years compared to the 2.6-fold increase in other types of publications could have played a role here [67]. Moreover, in 2019, 24% of the SRs available from PubMed were published in China [67] but were excluded from this review due to the lack of competence in reading these SRs, and it is difficult to know if our results would be similar if we have had included these.

In the last decade, there has been an increased interest in exploring yoga’s effectiveness in chronic pain. Yoga combines physical postures, breathing, and meditation intending to enhance physical and mental well-being [22, 58–60]. However, the use of yoga in chronic neck pain has been studied to a lesser extent. In our SR of SRs, yoga showed low-to-moderate certainty evidence for positive results on pain and disability in the short-term compared to other exercises as well as to non-exercise interventions. Even if based on a few SRs [22, 58–60] and with varying quality, this finding is in line with the network analysis of original studies [65]. The evidence of the few and highly overlapping reviews in our SR of SRs yielded a moderate level of certainty that yoga may be effective for neck pain and disability in the short-term. No studies reported long-term effects. The interventions were compared with several mixed interventions, which made it difficult to elucidate its actual comparison. The extreme heterogeneity among the yoga interventions in terms of style, the number of sessions, their length, frequency, and intensity, and the comparison groups were remarkable when summarizing the reviews. This makes the implication of our findings uncertain, and it is difficult to provide clear clinical guidance. Additionally, very few studies had studied the effects over time and only assessed the effects at post-intervention. In yoga, the mind–body relation is in focus. However, our review included only outcomes on the “body” without considering outcomes concerning the “mind”, such as quality of life and mood. Even if not investigated in the current SR of SRs, the reason for the positive effect reported for yoga might be the “mind” perspective.

The neck is affected in different ways by static positions such as those seen in office workers or by heavy loads on the arms [24, 25]. The neck can also be considered less robust than the lower back with the range of motion between the segments being larger than in the lower back [68]. Even if neck pain differs from lower back pain our results are in line with what we found summarizing the literature in a SR of SRs on various exercise types used in chronic low back pain, reporting that no exercise type seems to have a positive effect compared to any other, while there seems to be a positive effect compared to non-exercise interventions [27]. In addition, studies have shown that chronic, long-lasting, and recurrent pain sensations lead to changes in the nervous system such as increased peripheral and central sensitization that results in decreased motor function [69]. The improvement of disability that some of the included SRs reported after a training period could be explained by increased physiological functioning, such as increased muscle strength and endurance, increased range of motion, increased relaxation of tensed muscles, or lower pain intensity due to increased endorphin production. However, non-physiological concepts of pain treatment could also play an important role because pain is nowadays seen as a homeostatic emotion [70] in which pain is influenced by changes in the nervous system rather than by changes in tissues [71–74]. Psychological factors – including catastrophizing, anxiety, avoidance behavior, and depression – are also important in the processes of local and central sensitization, and these factors are all positively influenced by exercise and light physical activity [75, 76]. Thus, the use of exercise treatment in chronic pain conditions should be seen as a form of cognitive therapy where the goal is to modulate the feeling of pain and to modulate the patients’ thoughts and feelings regarding the pain, and not just to increase muscle strength and endurance [77]. This could explain why the choice of exercise type and dosage seems to be of less importance in the treatment of patients with chronic neck pain.

### Strengths and limitations

A strength of the present SR of SRs is that a large group of experienced researchers focused on this research question and followed the PRISMA recommendations as well as the recommendations from the Cochrane Back- and Neck group [29, 30]. We included SRs from several databases and thus base our results on a large study population. Furthermore, we did not limit our search and included SRs without any restrictions on publishing year, comparator group, or language. In addition, to our knowledge, this is the first SR of SRs on the effect of various exercise types used in chronic neck pain. A network analysis was recently published on the same topic but included original studies and thus missed out on the original SRs’ conclusions and the risk of bias affecting the certainty of the evidence [65].

Several limitations should be noted. The first is that our results are based on several SRs with a critically low to low quality (n = 13), which account for more than half of our included SRs. Another limitation is that we used data presented in the included SRs, without thoroughly checking if the data from the original RCTs were correct. Interestingly, we were astonished by the number of errors that were published in both SRs and MAs, when randomly checking some of the reported data to the original RCTs and extracting the data for the meta-analyses. We would therefore like to advocate for the highest accuracy when conducting systematic reviews and meta-analyses. Journal editors might, in addition, consider implying control mechanisms to avoid these kinds of errors. Our experience is that performing a peer review of SRs is very time-consuming, and we believe that most reviewers trust the tables and the meta-analyses maybe without checking them. Moreover, the AMSTAR-2 tool lacks the possibility to lower the quality of the included SRs based on this point. Due to the transparency of protocols, there is however low possibility of adding additional exclusion criteria during this process for these kinds of poorly conducted/reported trials.

Most of our included SRs were downgraded due to low quality and inconsistency in results. It might be considered that to have increased certainty of evidence for exercise types used in chronic neck pain we should have included only SRs with a moderate to high quality, thus reaching different conclusions. However, our aim was to include all SRs and MAs on exercises used in chronic neck pain without limitation. Another limitation, and a challenge when conducting a SR of SRs, is how to interpret the results of the included SRs because not all are clear on how participants and interventions are described or how the effects are measured. Some of our included SRs also included a variety of mixed interventions, for example, various exercise types conducted in the same intervention or exercises together with other interventions [26, 42, 58]. Some SRs also used their own definitions for the exercise types that were not used in the same way in other SRs [26]. Another challenge in summarizing the evidence in our SR of SRs is the large overlap shown for some of the exercise types. Motor control exercises, for example, had a high overlap of 26%, which means that several of the original studies were included in several of the SRs. Even so, the results based on the included SRs were somewhat ambiguous.

Chronic neck pain is a wide and heterogeneous diagnosis that might include, for example, patients who suffer from whiplash-associated disorders. We decided not to include SRs investigating patients suffering from whiplash-associated disorders because these often require a more multimodal approach instead of a single exercise intervention. However, we cannot rule out that some of our included SRs comprise such populations even if all defined their populations as having chronic neck pain.

Furthermore, we cannot say anything regarding the dose and duration of the various exercise types, which were heterogeneous in the various SRs, but defining the optimal dose was not the aim of our study. Based on the mechanisms that affect pain and disability levels as previously discussed, the dose and duration are important factors and should be investigated in future studies. In addition, we cannot rule out if a more pragmatic approach including other modalities in addition to the exercises investigated would have changed our results because such an approach was not the aim of our study.

Our results show that exercises have an overall positive effect on pain and disability compared with non-exercise interventions, at least in the short-term. However, in the intermediate/long-term there are varying results, and it remains unclear if the exercises studied in this review are effective when compared to non-exercise controls or to other exercises. Overall, the decision on what exercise type to use in the clinic should be in dialogue with the patient, which is the recommended way of working in a patient-centered way [78]. In addition, adherence to the exercises is seemingly important for a successful outcome [79, 80], but this was not investigated in the present SR of SRs.

Going forward, it is important that future SRs follow the recommendations on how to perform a SR with good quality using e.g. the PRISMA or the Cochrane group guidelines [29, 30] and that they also report the certainty of the evidence for the reader to be able to value the results [41]. Moreover, future original RCTs should preferably include larger cohorts and better-defined control groups so that a within-group comparison is feasible. Considering regression to the mean it is also important that there is a clear contrast between intervention and control group. All interventions and control groups should also be clearly described based on e.g. the template for Intervention and Replication (TIDieR) checklist [81]. Summarizing the literature in a SR of SRs has the advantage of being able to show that within an area of research, there are several SRs and MAs with low to critically low quality also based on original studies with a lower quality, thus affecting the overall evidence. The aim of a SR of SRs such as ours was to identify and appraise all published reviews in one area of interest and to describe their quality, summarize and compare their conclusions, and discuss the strengths of these conclusions [28]. This is important to highlight as many guidelines base their recommendation on how to manage a specific group of patients by summarizing the results of SRs.

## Conclusion

Overall, our findings show low to high certainty of evidence for positive effects on pain and disability of the various exercise types used in chronic neck pain compared to non-exercise interventions, at least in the short-term. Compared to other exercises MCE showed short-term effects on pain and disability levels while no such effects were shown for the other exercise types. What exercises to choose for the individual patient with chronic neck pain cannot be recommended from our results since we found no large differences between the exercise types studied here. Because the quality of the included SRs varied greatly, future SRs need to increase their methodological quality.

### Supplementary Information

**Additional file 1.** PRISMA statement of items to include when reporting a systematic review or meta-analysis.

**Additional file 2.** Inclusion and exclusion criteria according to PICO.

**Additional file 3.** Search strategy.

**Additional file 4.** Excluded papers based on full-text reading and reasons. 

**Additional file 5.** Meta-analyses based on data from the included SRs (MAs) for each exercise type.

## Data Availability

All data generated or analyzed during this study are included in this published article [and its supplementary information files].
