# Supplementary material for: Summarizing the effects of different exercise types in chronic neck pain – a systematic review and meta-analysis of systematic reviews
Source: BMC Musculoskelet Disord. 2023 Oct 12;24:806. doi: 10.1186/s12891-023-06930-9 (PMC10568903; doi:10.1186/s12891-023-06930-9)
Supplement: Supplementary file 2 — Additional file 2. Inclusion and exclusion criteria according to PICO. [file 12891_2023_6930_MOESM2_ESM.docx]

| **Additional file 2**: Inclusion and exclusion criteria according to PICO | | |
| --- | --- | --- |
|  | **Including criteria** | **Excluding criteria** |
| S (Setting) | At least 75% of the included studies should be RCT’s.  All languages |  |
| P (population) | At least 75% of the included studies should include adults between 18 and 70 years of age who suffered from chronic or recurrent unspecific CNP for a period of at least 12 weeks | Specific CNP, malign or systematic-caused pain, fractures, rheumatologic-caused, widespread chronic pain, multiple sclerosis or other neurologic diseases and neuropathic illnesses, or CNP due to psychiatric diagnoses. Whiplash-related CNP. |
| I (Intervention) | All different types of exercise treatments | Multimodal/multi or interdisciplinary rehabilitation, back schools, general physical activity recommendations, and occupational physical activity (graded activity). Studies were excluded if more than 75% of the included RCT included combinations of exercises modalities (e.g. MCE + resistance training vs resistance training alone). |
| C (Control) | All control groups |  |
| O (Outcome) | Pain and disability | Outcomes not relevant for the patient, such as health economics |
