# Supplementary material for: Summarizing the effects of different exercise types in chronic neck pain – a systematic review and meta-analysis of systematic reviews
Source: BMC Musculoskelet Disord. 2023 Oct 12;24:806. doi: 10.1186/s12891-023-06930-9 (PMC10568903; doi:10.1186/s12891-023-06930-9)
Supplement: Supplementary file 3 — Additional file 3. Search strategy. [file 12891_2023_6930_MOESM3_ESM.docx]

**Additional file 3:** Search strategy

1. Medline

| Interface: Ovid MEDLINE(R) and Epub Ahead of Print, In-Process & Other Non-Indexed Citations and Daily  Date of Search: 29 April 2022 Number of hits: 275  Comment: In Ovid, two or more words are automatically searched as phrases; i.e. no quotation marks are needed | Field labels   - exp/ = exploded MeSH term - / = non exploded MeSH term - .ti,ab,kf. = title, abstract and author keywords - adjx = within x words, regardless of order - * = truncation of word for alternate endings |
| --- | --- |
| \| **#** \| **Query** \| **Hits** \| \| --- \| --- \| --- \| \| 1 \| Neck Pain/ \| 7985 \| \| 2 \| (cervicalgi* or cervicodyni*).ti,ab,kf. \| 196 \| \| 3 \| exp Musculoskeletal Pain/ \| 6709 \| \| 4 \| (ache* or myalgi* or neuralgi* or pain*).ti,ab,kf. \| 829287 \| \| 5 \| or/3-4 \| 830805 \| \| 6 \| exp Neck/ \| 32462 \| \| 7 \| exp Cervical Vertebrae/ \| 42584 \| \| 8 \| (atlas or cervical disk or cervical spine or cervical vertebra* or neck*).ti,ab,kf. \| 301367 \| \| 9 \| or/6-8 \| 333733 \| \| 10 \| 5 and 9 \| 34582 \| \| 11 \| 1 or 2 or 10 \| 36588 \| \| 12 \| exp Exercise/ \| 230671 \| \| 13 \| exp Exercise Therapy/ \| 59428 \| \| 14 \| exp Exercise Movement Techniques/ \| 9664 \| \| 15 \| exp Sports/ \| 205505 \| \| 16 \| (aerobic* or aquatherap* or aqua therap* or bicycle* or bicycling or cycling or calisthenic* or callisthenic* or cardiopulmonary conditioning or climbing or danc* or exercise* or gi gong or gigong or gymnastic or hiit or hopping or hydrotherap* or isometric training or jogging or jumping or kinesiolog* or motion therap* or movement therap* or pilates or physical condition* or plyometric* or relaxation* or running or sport* or stretching or swim* or t'ai chi or tai or thai or taiji or taijiquan or taichi or treadmill or walk* or warm-up or water therap* or weight* lifting or lifting weight* or weightlifting or power lifting or water therap* or weight training or yoga).ti,ab,kf. \| 962021 \| \| 17 \| (physical* adj2 (activ* or training)).ti,ab,kf. \| 151129 \| \| 18 \| ((anaerobic or cardio* or circuit or interval* or motor control or muscle* or resistance* or strength*) adj2 (program* or training)).ti,ab,kf. \| 33605 \| \| 19 \| or/12-18 \| 1154902 \| \| 20 \| 11 and 19 \| 4244 \| \| 21 \| Meta-analysis.pt. \| 159501 \| \| 22 \| Meta-analysis as topic/ \| 21314 \| \| 23 \| Systematic Reviews as Topic/ \| 8241 \| \| 24 \| ((systematic or umbrella or scoping) adj3 review).ti. \| 197748 \| \| 25 \| (meta analys* or metaanalys* or meta synthes* or metasynthes*).ti. \| 152724 \| \| 26 \| "review of reviews".ti. \| 338 \| \| 27 \| Systematic Review.pt. \| 194702 \| \| 28 \| or/21-27 \| 355650 \| \| 29 \| 20 and 28 \| 275 \| | |

2. Embase

| Interface: embase.com  Date of Search: 29 April 2022  Number of hits: 487  Comment: Emtree is the controlled vocabulary in Embase | Field labels   - /exp = exploded Emtree term - /de = non exploded Emtree term - ti,ab,kw = title, abstract and author keywords - NEAR/x = within x words, regardless of order - * = truncation of word for alternate endings |
| --- | --- |
| \| **#** \| **Query** \| **Hits** \| \| --- \| --- \| --- \| \| #1 \| 'neck pain'/exp \| 27,678 \| \| #2 \| cervicalgi*:ti,ab,kw OR cervicodyni*:ti,ab,kw \| 320 \| \| #3 \| 'musculoskeletal pain'/exp \| 173,573 \| \| #4 \| ache*:ti,ab,kw OR myalgi*:ti,ab,kw OR neuralgi*:ti,ab,kw OR pain*:ti,ab,kw \| 1,227,322 \| \| #5 \| #3 OR #4 \| 1,285,426 \| \| #6 \| 'neck'/exp \| 85,752 \| \| #7 \| 'cervical spine'/exp \| 46,209 \| \| #8 \| atlas:ti,ab,kw OR 'cervical disk':ti,ab,kw OR 'cervical spine':ti,ab,kw OR 'cervical vertebra*':ti,ab,kw OR neck*:ti,ab,kw \| 424,632 \| \| #9 \| #6 OR #7 OR #8 \| 462,527 \| \| #10 \| #5 AND #9 \| 58,170 \| \| #11 \| #1 OR #2 OR #10 \| 66,882 \| \| #12 \| 'exercise'/exp \| 402,494 \| \| #13 \| 'kinesiotherapy'/exp \| 93,997 \| \| #14 \| 'sport'/exp \| 198,699 \| \| #15 \| aerobic*:ti,ab,kw OR aquatherap*:ti,ab,kw OR 'aqua therap*':ti,ab,kw OR bicycle*:ti,ab,kw OR bicycling:ti,ab,kw OR cycling:ti,ab,kw OR calisthenic*:ti,ab,kw OR callisthenic*:ti,ab,kw OR 'cardiopulmonary conditioning':ti,ab,kw OR climbing:ti,ab,kw OR danc*:ti,ab,kw OR exercise*:ti,ab,kw OR 'qi gong':ti,ab,kw OR qigong:ti,ab,kw OR gymnastic:ti,ab,kw OR hiit:ti,ab,kw OR hopping:ti,ab,kw OR hydrotherap*:ti,ab,kw OR 'isometric training':ti,ab,kw OR jogging:ti,ab,kw OR jumping:ti,ab,kw OR kinesiolog*:ti,ab,kw OR 'motion therap*':ti,ab,kw OR 'movement therap*':ti,ab,kw OR pilates:ti,ab,kw OR 'physical condition*':ti,ab,kw OR plyometric*:ti,ab,kw OR relaxation*:ti,ab,kw OR running:ti,ab,kw OR sport*:ti,ab,kw OR stretching:ti,ab,kw OR swim*:ti,ab,kw OR 'tai chi':ti,ab,kw OR taiji:ti,ab,kw OR taijiquan:ti,ab,kw OR taichi:ti,ab,kw OR treadmill:ti,ab,kw OR walk*:ti,ab,kw OR 'warm up':ti,ab,kw OR 'weight lifting':ti,ab,kw OR 'lifting weight*':ti,ab,kw OR weightlifting:ti,ab,kw OR 'power lifting':ti,ab,kw OR 'water therap*':ti,ab,kw OR 'weight training':ti,ab,kw OR yoga:ti,ab,kw \| 1,184,046 \| \| #16 \| (physical* NEAR/2 (activ* OR training)):ti,ab,kw \| 204,812 \| \| #17 \| ((anaerobic OR cardio* OR circuit OR interval* OR 'motor control' OR muscle* OR resistance* OR strength*) NEAR/2 (program* OR training)):ti,ab,kw \| 44,001 \| \| #18 \| #12 OR #13 OR #14 OR #15 OR #16 OR #17 \| 1,475,264 \| \| #19 \| #11 AND #18 \| 7,877 \| \| #20 \| 'systematic review'/exp \| 342,609 \| \| #21 \| 'systematic review (topic)'/exp \| 28,623 \| \| #22 \| 'meta analysis'/exp \| 244,142 \| \| #23 \| 'meta analysis (topic)'/exp \| 49,124 \| \| #24 \| ((systematic OR umbrella OR scoping) NEAR/3 review):ti \| 231,248 \| \| #25 \| 'meta analys*':ti OR metaanalys*:ti OR 'meta synthes*':ti OR metasynthes*:ti \| 187,702 \| \| #26 \| 'review of reviews':ti \| 348 \| \| #27 \| #20 OR #21 OR #22 OR #23 OR #24 OR #25 OR #26 \| 564,091 \| \| #28 \| #19 AND #27 \| 487 \| | |

3. Cochrane Library

| Interface: Wiley  Date of Search: 29 April 2022  Number of hits: 35 | Field labels   - ti,ab,kw = title, abstract and author keywords - NEAR/x = within x words, regardless of order - * = truncation of word for alternate endings |
| --- | --- |
| \| **#** \| **Search** \| **Hits** \| \| --- \| --- \| --- \| \| #1 \| MeSH descriptor: [Neck Pain] explode all trees \| 1501 \| \| #2 \| (cervicalgi* or cervicodyni*):ti,ab,kw \| 84 \| \| #3 \| MeSH descriptor: [Musculoskeletal Pain] explode all trees \| 1182 \| \| #4 \| (ache* or myalgi* or neuralgi* or pain*):ti,ab,kw \| 210919 \| \| #5 \| #3 OR #4 \| 210919 \| \| #6 \| MeSH descriptor: [Neck] explode all trees \| 564 \| \| #7 \| MeSH descriptor: [Cervical Vertebrae] explode all trees \| 1027 \| \| #8 \| (atlas or cervical disk or cervical spine or cervical vertebra* or neck*):ti,ab,kw \| 28866 \| \| #9 \| #6 OR #7 OR #8 \| 28868 \| \| #10 \| #5 AND #9 \| 8758 \| \| #11 \| #1 OR #2 OR #10 \| 8768 \| \| #12 \| MeSH descriptor: [Exercise] explode all trees \| 28159 \| \| #13 \| MeSH descriptor: [Exercise Therapy] explode all trees \| 16029 \| \| #14 \| MeSH descriptor: [Exercise Movement Techniques] explode all trees \| 2474 \| \| #15 \| MeSH descriptor: [Sports] explode all trees \| 17073 \| \| #16 \| (aerobic* or aquatherap* or "aqua therap*" or bicycle* or bicycling or cycling or calisthenic* or callisthenic* or "cardiopulmonary conditioning" or climbing or danc* or exercise* or "qi gong" or qigong or gymnastic or hiit or hopping or hydrotherap* or "isometric training" or jogging or jumping or kinesiolog* or "motion therap*" or "movement therap*" or pilates or "physical condition*" or plyometric* or relaxation* or running or sport* or stretching or swim* or "tai chi" or tai or thai or taiji or taijiquan or taichi or treadmill or walk* or warm-up or "water therap*" or "weight* lifting" or "lifting weight*" or weightlifting or "power lifting" or "water therap*" or "weight training" or yoga):ti,ab,kw \| 166719 \| \| #17 \| (physical* NEAR/2 (activ* or training)):ti,ab,kw \| 40613 \| \| #18 \| ((anaerobic or cardio* or circuit or interval* or "motor control" or muscle* or resistance* or strength*) NEAR/2 (program* or training)):ti,ab,kw \| 23713 \| \| #19 \| #12 OR #13 OR #14 OR #15 OR #16 OR #17 OR #18 \| 187330 \| \| #20 \| #11 AND #19 \| 2554 \| \| #21 \| #20 in Cochrane Reviews, Cochrane Protocols \| 35 \| | |

4. Web of Science Core Collection

| Interface: Clarivate Analytics  Editions = A&HCI , ESCI , SCI-EXPANDED , SSCI  Date of Search: 29 April 2022  Number of hits: 3,400 | Field labels   - TS/Topic = title, abstract, author keywords and Keywords Plus - NEAR/x = within x words, regardless of order - * = truncation of word for alternate endings   Note: the *Exact search*-function was used for all the searches |
| --- | --- |
| \| **#** \| **Query** \| **Hits** \| \| --- \| --- \| --- \| \| 1 \| (cervicalgi* or cervicodyni*) (Topic) \| 145 \| \| 2 \| (ache* or myalgi* or neuralgi* or pain*) (Topic) \| 893,111 \| \| 3 \| (atlas or cervical disk or cervical spine or cervical vertebra* or neck*) (Topic) \| 370,236 \| \| 4 \| #3 AND #2 \| 35,838 \| \| 5 \| (aerobic* or aquatherap* or "aqua therap*" or bicycle* or bicycling or cycling or calisthenic* or callisthenic* or "cardiopulmonary conditioning" or climbing or danc* or exercise* or "gi gong" or gigong or gymnastic or hiit or hopping or hydrotherap* or "isometric training" or jogging or jumping or kinesiolog* or "motion therap*" or "movement therap*" or pilates or "physical condition*" or plyometric* or relaxation* or running or sport* or stretching or swim* or t'ai chi or tai or thai or taiji or taijiquan or taichi or treadmill or walk* or warm-up or "water therap*" or "weight* lifting" or "lifting weight*" or weightlifting or "power lifting" or "water therap*" or "weight training" or yoga) (Topic) \| 3,596,801 \| \| 6 \| (physical* NEAR/2 (activ* or training)) (Topic) \| 236,18 \| \| 7 \| ((anaerobic or cardio* or circuit or interval* or "motor control" or muscle* or resistance* or strength*) NEAR/2 (program* or training)) (Topic) \| 54,297 \| \| 8 \| #7 OR #6 OR #5 \| 3,742,672 \| \| 9 \| #1 OR #4 \| 35,93 \| \| 10 \| #9 AND #8 \| 5,525 \| \| 11 \| ((systematic or umbrella or scoping) NEAR/3 review) (Title) \| 231,811 \| \| 12 \| ("meta analys*" or metaanalys* or meta synthes* or metasynthes*) (Title) \| 185,073 \| \| 13 \| "review of reviews" (Title) \| 426 \| \| 14 \| #13 OR #12 OR #11 \| 331,4 \| \| 15 \| #9 AND #8 and Review Articles (Document Types) \| 677 \| \| 16 \| #10 AND #14 \| 351 \| \| 17 \| #16 OR #15 \| 718 \| | |

5. SportDiscus

| Interface: Ebsco  Date of Search: 29 April 2022  Number of hits: 82 | Field labels   - SU = word in thesaurus term - DE = exact thesaurus term - TI = title - AB = abstract - KW = author’s keyword - Nx = within x words, regardless of order - * = truncation of word for alternate endings |
| --- | --- |
| \| **#** \| **Query** \| **Results** \| \| --- \| --- \| --- \| \| 1 \| SU "Neck pain" \| 1,466 \| \| 2 \| TI ( cervicalg* OR cervicodyn* ) OR AB ( cervicalg* OR cervicodyn* ) OR KW ( cervicalg* OR cervicodyn* ) \| 48 \| \| 3 \| DE "NECK" \| 1,236 \| \| 4 \| DE "NECK muscles" OR DE "SCALENE muscles" OR DE "SPLENIUS muscles" OR DE "STERNOCLEIDOMASTOID muscle" \| 414 \| \| 5 \| DE "CERVICAL vertebrae" \| 1,809 \| \| 6 \| DE "NECK physiology" \| 114 \| \| 7 \| S3 OR S4 OR S5 OR S6 \| 3,304 \| \| 8 \| DE "PAIN" \| 10,276 \| \| 9 \| DE "Myalgia" \| 1,779 \| \| 10 \| TI ( ache* OR myalgi* OR neuralg* OR pain* ) OR AB ( ache* OR myalgi* OR neuralg* OR pain* ) OR KW ( ache* OR myalgi* OR neuralg* OR pain* ) \| 64,942 \| \| 11 \| S8 OR S9 OR S10 \| 67,827 \| \| 12 \| S7 AND S11 \| 929 \| \| 13 \| TI ( ( atlas or "cervical disk*" OR "cervical spine" OR "cervical vertebra*" OR neck OR scalene OR "splenius muscle*" OR sternocleidomastoid ) N3 (ache* OR myalgi* OR neuralgi* OR pain) ) OR AB ( ( atlas or "cervical disk*" OR "cervical spine" OR "cervical vertebra*" OR neck OR scalene OR "splenius muscle*" OR sternocleidomastoid ) N3 (ache* OR myalgi* OR neuralgi* OR pain) ) OR KW ( ( atlas or "cervical disk*" OR "cervical spine" OR "cervical vertebra*" OR neck OR scalene OR "splenius muscle*" OR sternocleidomastoid ) N3 (ache* OR myalgi* OR neuralgi* OR pain) ) \| 2,602 \| \| 14 \| S1 OR S2 OR S12 OR S13 \| 3,238 \| \| 15 \| DE "PHYSICAL activity" \| 22,649 \| \| 16 \| SU exercise* \| 153,790 \| \| 17 \| TI ( (aerobic* or aquatherap* or "aqua therap*" or bicycle* or bicycling or cycling or calisthenic* or callisthenic* or "cardiopulmonary conditioning" or climbing or danc* or exercise* or "gi gong" or gigong or gymnastic or hiit or hopping or hydrotherap* or "isometric training" or jogging or jumping or kinesiolog* or "motion therap*" or "movement therap*" or pilates or "physical condition*" or plyometric* or relaxation* or running or sport* or stretching or swim* or "tai chi" or tai or thai or taiji or taijiquan or taichi or treadmill or walk* or warm-up or "water therap*" or "weight* lifting" or "lifting weight*" or weightlifting or "power lifting" or "water therap*" or "weight training" or yoga) ) OR AB ( (aerobic* or aquatherap* or "aqua therap*" or bicycle* or bicycling or cycling or calisthenic* or callisthenic* or "cardiopulmonary conditioning" or climbing or danc* or exercise* or "gi gong" or gigong or gymnastic or hiit or hopping or hydrotherap* or "isometric training" or jogging or jumping or kinesiolog* or "motion therap*" or "movement therap*" or pilates or "physical condition*" or plyometric* or relaxation* or running or sport* or stretching or swim* or "tai chi" or tai or thai or taiji or taijiquan or taichi or treadmill or walk* or warm-up or "water therap*" or "weight* lifting" or "lifting weight*" or weightlifting or "power lifting" or "water therap*" or "weight training" or yoga) ) OR KW ( (aerobic* or aquatherap* or "aqua therap*" or bicycle* or bicycling or cycling or calisthenic* or callisthenic* or "cardiopulmonary conditioning" or climbing or danc* or exercise* or "gi gong" or gigong or gymnastic or hiit or hopping or hydrotherap* or "isometric training" or jogging or jumping or kinesiolog* or "motion therap*" or "movement therap*" or pilates or "physical condition*" or plyometric* or relaxation* or running or sport* or stretching or swim* or "tai chi" or tai or thai or taiji or taijiquan or taichi or treadmill or walk* or warm-up or "water therap*" or "weight* lifting" or "lifting weight*" or weightlifting or "power lifting" or "water therap*" or "weight training" or yoga) ) \| 696,822 \| \| 18 \| DE "SPORTS" OR DE "AERODYNAMICS in sports" OR DE "AERONAUTICAL sports" OR OR DE "AQUATIC sports" OR DE "BALL games" OR DE "BALLISTICS in sports" OR DE "BASEBALL" OR DE "COMBAT sports" OR DE "CONTACT sports" OR DE "CROSS-training (Sports)" OR DE "DISC golf" OR DE "ENDURANCE sports" OR DE "EXTREME sports" OR DE "FANTASY sports" OR DE "GYMNASTICS" OR DE "HOCKEY" OR DE "PARKOUR" OR DE "RACKET games" OR DE "RECREATIONAL sports" OR DE "ROLLER skating" OR DE "SKATEBOARDING" OR DE "SOFTBALL" OR DE "TEAM sports" \| 180,087 \| \| 19 \| TI ( physical* N2 (activ* or training) ) OR AB ( physical* N2 (activ* or training) ) OR KW ( physical* N2 (activ* or training) ) \| 65,770 \| \| 20 \| TI ( (anaerobic or cardio* or circuit or interval* or "motor control" or muscle* or resistance* or strength*) N2 (program* or training) ) OR AB ( (anaerobic or cardio* or circuit or interval* or "motor control" or muscle* or resistance* or strength*) N2 (program* or training) ) OR KW ( (anaerobic or cardio* or circuit or interval* or "motor control" or muscle* or resistance* or strength*) N2 (program* or training) ) \| 30,364 \| \| 21 \| S15 OR S16 OR S17 OR S18 OR S19 OR S20 \| 855,157 \| \| 22 \| S14 AND S21 \| 1,056 \| \| 23 \| TI ((systematic or umbrella or scoping) N2 review) \| 49,437 \| \| 24 \| TI ("meta analys*" or metaanalys* or meta synthes* or metasynthes*) \| 37,250 \| \| 25 \| TI ("review of reviews") \| 73 \| \| 26 \| S23 OR S24 OR S25 OR S26 \| 83,250 \| \| 27 \| S22 AND S26 \| 82 \| | |

**Updated search 23-06-28**

Databases:

1. Medline (Ovid)
2. Embase (embase.com)
3. Cochrane (Wiley)
4. Web of Science (Clarivate)
5. SportDiscus (Ebsco)

Total number of hits:

- Before deduplication: 1619 (2023 whitout Sportdiscus) + 82 (Sportdiscus in 2022) = 1701
- After deduplication: 1,049 (2022) + 170 (upd 2023) = 1219

Comments:

Deduplication based on the method described in:
Bramer, W. M., Giustini, D., de Jonge, G. B., Holland, L., & Bekhuis, T. (2016). De-duplication of database search results for systematic reviews in EndNote. *Journal of the Medical Library Association: JMLA*, 104(3), 240–243. doi:10.3163/1536-5050.104.3.014

One final, extra step was added to compare DOIs.

1. Medline

| Interface: Ovid MEDLINE(R) ALL 1946 to June 27, 2023  Date of Search: 28 June 2023  Number of hits: 322  Comment: In Ovid, two or more words are automatically searched as phrases; i.e. no quotation marks are needed | Field labels   - exp/ = exploded MeSH term - / = non exploded MeSH term - .ti,ab,kf. = title, abstract and author keywords - adjx = within x words, regardless of order - * = truncation of word for alternate endings |
| --- | --- |
| Database(s): **Ovid MEDLINE(R) ALL**1946 to June 27, 2023 Search Strategy:   \| **#** \| **Searches** \| **Results** \| \| --- \| --- \| --- \| \| 1 \| Neck Pain/ \| 8501 \| \| 2 \| (cervicalgi* or cervicodyni*).ti,ab,kf. \| 221 \| \| 3 \| exp Musculoskeletal Pain/ \| 7449 \| \| 4 \| (ache* or myalgi* or neuralgi* or pain*).ti,ab,kf. \| 893872 \| \| 5 \| or/3-4 \| 895480 \| \| 6 \| exp Neck/ \| 33642 \| \| 7 \| exp Cervical Vertebrae/ \| 43920 \| \| 8 \| (atlas or cervical disk or cervical spine or cervical vertebra* or neck*).ti,ab,kf. \| 326799 \| \| 9 \| or/6-8 \| 359807 \| \| 10 \| 5 and 9 \| 37490 \| \| 11 \| 1 or 2 or 10 \| 39533 \| \| 12 \| exp Exercise/ \| 246039 \| \| 13 \| exp Exercise Therapy/ \| 63214 \| \| 14 \| exp Exercise Movement Techniques/ \| 10272 \| \| 15 \| exp Sports/ \| 215925 \| \| 16 \| (aerobic* or aquatherap* or aqua therap* or bicycle* or bicycling or cycling or calisthenic* or callisthenic* or cardiopulmonary conditioning or climbing or danc* or exercise* or gi gong or gigong or gymnastic or hiit or hopping or hydrotherap* or isometric training or jogging or jumping or kinesiolog* or motion therap* or movement therap* or pilates or physical condition* or plyometric* or relaxation* or running or sport* or stretching or swim* or t'ai chi or tai or thai or taiji or taijiquan or taichi or treadmill or walk* or warm-up or water therap* or weight* lifting or lifting weight* or weightlifting or power lifting or water therap* or weight training or yoga).ti,ab,kf. \| 1035354 \| \| 17 \| (physical* adj2 (activ* or training)).ti,ab,kf. \| 167758 \| \| 18 \| ((anaerobic or cardio* or circuit or interval* or motor control or muscle* or resistance* or strength*) adj2 (program* or training)).ti,ab,kf. \| 37378 \| \| 19 \| or/12-18 \| 1241173 \| \| 20 \| 11 and 19 \| 4657 \| \| 21 \| Meta-analysis.pt. \| 183235 \| \| 22 \| Meta-analysis as topic/ \| 22411 \| \| 23 \| Systematic Reviews as Topic/ \| 10718 \| \| 24 \| ((systematic or umbrella or scoping) adj3 review).ti. \| 244737 \| \| 25 \| (meta analys* or metaanalys* or meta synthes* or metasynthes*).ti. \| 181364 \| \| 26 \| "review of reviews".ti. \| 403 \| \| 27 \| Systematic Review.pt. \| 231907 \| \| 28 \| or/21-27 \| 418359 \| \| 29 \| 20 and 28 \| 322 \| | |

2. Embase

| Interface: embase.com  Date of Search: 29 April 2022  Number of hits: 487  Comment: Emtree is the controlled vocabulary in Embase | Field labels   - /exp = exploded Emtree term - /de = non exploded Emtree term - ti,ab,kw = title, abstract and author keywords - NEAR/x = within x words, regardless of order - * = truncation of word for alternate endings |
| --- | --- |
| **No.**  **Query**  **Results**  **574**  **#28**  **#19** AND **#27**  **662,652**  **#27**  **#20** OR **#21** OR **#22** OR **#23** OR **#24** OR **#25** OR **#26**  **418**  **#26**  **'review of reviews'**:ti  **220,995**  **#25**  **'meta analys*'**:ti OR **metaanalys***:ti OR **'meta synthes*'**:ti OR **metasynthes***:ti  **283,656**  **#24**  ((**systematic** OR **umbrella** OR **scoping**) NEAR/3 **review**):ti  **52,775**  **#23**  **'meta analysis (topic)'**/exp  **283,493**  **#22**  **'meta analysis'**/exp  **31,853**  **#21**  **'systematic review (topic)'**/exp  **417,422**  **#20**  **'systematic review'**/exp  **8,699**  **#19**  **#11** AND **#18**  **1,589,000**  **#18**  **#12** OR **#13** OR **#14** OR **#15** OR **#16** OR **#17**  **48,757**  **#17**  ((**anaerobic** OR **cardio*** OR **circuit** OR **interval*** OR **'motor control'** OR **muscle*** OR **resistance*** OR **strength***) NEAR/2 (**program*** OR **training**)):ti,ab,kw  **226,061**  **#16**  (**physical*** NEAR/2 (**activ*** OR **training**)):ti,ab,kw  **1,273,064**  **#15**  **aerobic***:ti,ab,kw OR **aquatherap***:ti,ab,kw OR **'aqua therap*'**:ti,ab,kw OR **bicycle***:ti,ab,kw OR **bicycling**:ti,ab,kw OR **cycling**:ti,ab,kw OR **calisthenic***:ti,ab,kw OR **callisthenic***:ti,ab,kw OR **'cardiopulmonary conditioning'**:ti,ab,kw OR **climbing**:ti,ab,kw OR **danc***:ti,ab,kw OR **exercise***:ti,ab,kw OR **'qi gong'**:ti,ab,kw OR **qigong**:ti,ab,kw OR **gymnastic**:ti,ab,kw OR **hiit**:ti,ab,kw OR **hopping**:ti,ab,kw OR **hydrotherap***:ti,ab,kw OR **'isometric training'**:ti,ab,kw OR **jogging**:ti,ab,kw OR **jumping**:ti,ab,kw OR **kinesiolog***:ti,ab,kw OR **'motion therap*'**:ti,ab,kw OR **'movement therap*'**:ti,ab,kw OR **pilates**:ti,ab,kw OR **'physical condition*'**:ti,ab,kw OR **plyometric***:ti,ab,kw OR **relaxation***:ti,ab,kw OR **running**:ti,ab,kw OR **sport***:ti,ab,kw OR **stretching**:ti,ab,kw OR **swim***:ti,ab,kw OR **'tai chi'**:ti,ab,kw OR **taiji**:ti,ab,kw OR **taijiquan**:ti,ab,kw OR **taichi**:ti,ab,kw OR **treadmill**:ti,ab,kw OR **walk***:ti,ab,kw OR **'warm up'**:ti,ab,kw OR **'weight lifting'**:ti,ab,kw OR **'lifting weight*'**:ti,ab,kw OR **weightlifting**:ti,ab,kw OR **'power lifting'**:ti,ab,kw OR **'water therap*'**:ti,ab,kw OR **'weight training'**:ti,ab,kw OR **yoga**:ti,ab,kw  **215,448**  **#14**  **'sport'**/exp  **102,220**  **#13**  **'kinesiotherapy'**/exp  **438,745**  **#12**  **'exercise'**/exp  **73,123**  **#11**  **#1** OR **#2** OR **#10**  **63,383**  **#10**  **#5** AND **#9**  **502,705**  **#9**  **#6** OR **#7** OR **#8**  **461,497**  **#8**  **atlas**:ti,ab,kw OR **'cervical disk'**:ti,ab,kw OR **'cervical spine'**:ti,ab,kw OR **'cervical vertebra*'**:ti,ab,kw OR **neck***:ti,ab,kw  **49,057**  **#7**  **'cervical spine'**/exp  **93,177**  **#6**  **'neck'**/exp  **1,389,091**  **#5**  **#3** OR **#4**  **1,326,018**  **#4**  **ache***:ti,ab,kw OR **myalgi***:ti,ab,kw OR **neuralgi***:ti,ab,kw OR **pain***:ti,ab,kw  **189,001**  **#3**  **'musculoskeletal pain'**/exp  **363**  **#2**  **cervicalgi***:ti,ab,kw OR **cervicodyni***:ti,ab,kw  **30,694**  **#1**  **'neck pain'**/exp | |

3. Cochrane Library

| Interface: Wiley  Date of Search: 28 June 2023  Number of hits: 35 | Field labels   - ti,ab,kw = title, abstract and author keywords - NEAR/x = within x words, regardless of order - * = truncation of word for alternate endings |
| --- | --- |
| ID Search Hits  #1 [mh ^"Neck Pain"] 1994  #2 (cervicalgi*:ti,ab,kw OR cervicodyni*:ti,ab,kw) 100  #3 [mh "Musculoskeletal Pain"] 2041  #4 (ache*:ti,ab,kw OR myalgi*:ti,ab,kw OR neuralgi*:ti,ab,kw OR pain*:ti,ab,kw) 237698  #5 #3 OR #4 237698  #6 [mh Neck] 964  #7 [mh "Cervical Vertebrae"] 1245  #8 (atlas:ti,ab,kw OR "cervical disk":ti,ab,kw OR "cervical spine":ti,ab,kw OR ("cervical" NEXT vertebra*):ti,ab,kw OR neck*:ti,ab,kw) 31624  #9 #6 OR #7 OR #8 31634  #10 #5 AND #9 9946  #11 #1 OR #2 OR #10 9959  #12 [mh Exercise] 38147  #13 [mh "Exercise Therapy"] 19513  #14 [mh "Exercise Movement Techniques"] 3191  #15 [mh Sports] 20832  #16 (aerobic*:ti,ab,kw OR aquatherap*:ti,ab,kw OR ("aqua" NEXT therap*):ti,ab,kw OR bicycle*:ti,ab,kw OR bicycling:ti,ab,kw OR cycling:ti,ab,kw OR calisthenic*:ti,ab,kw OR callisthenic*:ti,ab,kw OR "cardiopulmonary conditioning":ti,ab,kw OR climbing:ti,ab,kw OR danc*:ti,ab,kw OR exercise*:ti,ab,kw OR "gi gong":ti,ab,kw OR gigong:ti,ab,kw OR gymnastic:ti,ab,kw OR hiit:ti,ab,kw OR hopping:ti,ab,kw OR hydrotherap*:ti,ab,kw OR "isometric training":ti,ab,kw OR jogging:ti,ab,kw OR jumping:ti,ab,kw OR kinesiolog*:ti,ab,kw OR ("motion" NEXT therap*):ti,ab,kw OR ("movement" NEXT therap*):ti,ab,kw OR pilates:ti,ab,kw OR ("physical" NEXT condition*):ti,ab,kw OR plyometric*:ti,ab,kw OR relaxation*:ti,ab,kw OR running:ti,ab,kw OR sport*:ti,ab,kw OR stretching:ti,ab,kw OR swim*:ti,ab,kw OR "t'ai chi":ti,ab,kw OR tai:ti,ab,kw OR thai:ti,ab,kw OR taiji:ti,ab,kw OR taijiquan:ti,ab,kw OR taichi:ti,ab,kw OR treadmill:ti,ab,kw OR walk*:ti,ab,kw OR warm-up:ti,ab,kw OR ("water" NEXT therap*):ti,ab,kw OR (weight* NEXT "lifting"):ti,ab,kw OR ("lifting" NEXT weight*):ti,ab,kw OR weightlifting:ti,ab,kw OR "power lifting":ti,ab,kw OR ("water" NEXT therap*):ti,ab,kw OR "weight training":ti,ab,kw OR yoga:ti,ab,kw) 190422  #17 (physical*:ti,ab,kw NEAR/2 (activ*:ti,ab,kw OR training:ti,ab,kw)) 46910  #18 ((anaerobic:ti,ab,kw OR cardio*:ti,ab,kw OR circuit:ti,ab,kw OR interval*:ti,ab,kw OR "motor control":ti,ab,kw OR muscle*:ti,ab,kw OR resistance*:ti,ab,kw OR strength*:ti,ab,kw) NEAR/2 (program*:ti,ab,kw OR training:ti,ab,kw)) 27549  #19 #12 OR #13 OR #14 OR #15 OR #16 OR #17 OR #18 213340  #20 #11 AND #19 3069 | |

4. Web of Science Core Collection

| Interface: Clarivate Analytics  Editions = A&HCI , ESCI , SCI-EXPANDED , SSCI  Date of Search: 28 June 2023  Number of hits: 775 | Field labels   - TS/Topic = title, abstract, author keywords and Keywords Plus - NEAR/x = within x words, regardless of order - * = truncation of word for alternate endings   Note: the *Exact search*-function was used for all the searches |
| --- | --- |
| \| # \| Search Query \| Results \| \| --- \| --- \| --- \| \| 1 \| TS=((cervicalgi* or cervicodyni*) ) \| 171 \| \| 2 \| TS=((ache* or myalgi* or neuralgi* or pain*) ) \| 963918 \| \| 3 \| TS=((atlas or cervical disk or cervical spine or cervical vertebra* or neck*) ) \| 399799 \| \| 4 \| #3 AND #2 \| 38930 \| \| 5 \| TS=((aerobic* or aquatherap* or "aqua therap*" or bicycle* or bicycling or cycling or calisthenic* or callisthenic* or "cardiopulmonary conditioning" or climbing or danc* or exercise* or "gi gong" or gigong or gymnastic or hiit or hopping or hydrotherap* or "isometric training" or jogging or jumping or kinesiolog* or "motion therap*" or "movement therap*" or pilates or "physical condition*" or plyometric* or relaxation* or running or sport* or stretching or swim* or t'ai chi or tai or thai or taiji or taijiquan or taichi or treadmill or walk* or warm-up or "water therap*" or "weight* lifting" or "lifting weight*" or weightlifting or "power lifting" or "water therap*" or "weight training" or yoga) ) \| 2083410 \| \| 6 \| TS=((physical* NEAR/2 (activ* or training)) ) \| 261114 \| \| 7 \| TS=(((anaerobic or cardio* or circuit or interval* or "motor control" or muscle* or resistance* or strength*) NEAR/2 (program* or training)) ) \| 56120 \| \| 8 \| #7 OR #6 OR #5 \| 2245751 \| \| 9 \| #1 OR #4 \| 39034 \| \| 10 \| #9 AND #8 \| 5570 \| \| 11 \| TI=(((systematic or umbrella or scoping) NEAR/3 review) ) \| 282704 \| \| 12 \| TI=(("meta analys*" or metaanalys* or meta synthes* or metasynthes*) ) \| 218047 \| \| 13 \| TI=("review of reviews" ) \| 499 \| \| 14 \| #13 OR #12 OR #11 \| 394080 \| \| 15 \| (#9 AND #8) AND (DT==("REVIEW")) \| 731 \| \| 16 \| #10 AND #14 \| 414 \| \| 17 \| #16 OR #15 \| 775 \| | |

5. SportDiscus

| Interface: Ebsco  Date of Search: 29 April 2022  Number of hits: 82 | Field labels   - SU = word in thesaurus term - DE = exact thesaurus term - TI = title - AB = abstract - KW = author’s keyword - Nx = within x words, regardless of order - * = truncation of word for alternate endings |
| --- | --- |
| \| **#** \| **Query** \| **Results** \| \| --- \| --- \| --- \| \| 1 \| SU "Neck pain" \| 1,466 \| \| 2 \| TI ( cervicalg* OR cervicodyn* ) OR AB ( cervicalg* OR cervicodyn* ) OR KW ( cervicalg* OR cervicodyn* ) \| 48 \| \| 3 \| DE "NECK" \| 1,236 \| \| 4 \| DE "NECK muscles" OR DE "SCALENE muscles" OR DE "SPLENIUS muscles" OR DE "STERNOCLEIDOMASTOID muscle" \| 414 \| \| 5 \| DE "CERVICAL vertebrae" \| 1,809 \| \| 6 \| DE "NECK physiology" \| 114 \| \| 7 \| S3 OR S4 OR S5 OR S6 \| 3,304 \| \| 8 \| DE "PAIN" \| 10,276 \| \| 9 \| DE "Myalgia" \| 1,779 \| \| 10 \| TI ( ache* OR myalgi* OR neuralg* OR pain* ) OR AB ( ache* OR myalgi* OR neuralg* OR pain* ) OR KW ( ache* OR myalgi* OR neuralg* OR pain* ) \| 64,942 \| \| 11 \| S8 OR S9 OR S10 \| 67,827 \| \| 12 \| S7 AND S11 \| 929 \| \| 13 \| TI ( ( atlas or "cervical disk*" OR "cervical spine" OR "cervical vertebra*" OR neck OR scalene OR "splenius muscle*" OR sternocleidomastoid ) N3 (ache* OR myalgi* OR neuralgi* OR pain) ) OR AB ( ( atlas or "cervical disk*" OR "cervical spine" OR "cervical vertebra*" OR neck OR scalene OR "splenius muscle*" OR sternocleidomastoid ) N3 (ache* OR myalgi* OR neuralgi* OR pain) ) OR KW ( ( atlas or "cervical disk*" OR "cervical spine" OR "cervical vertebra*" OR neck OR scalene OR "splenius muscle*" OR sternocleidomastoid ) N3 (ache* OR myalgi* OR neuralgi* OR pain) ) \| 2,602 \| \| 14 \| S1 OR S2 OR S12 OR S13 \| 3,238 \| \| 15 \| DE "PHYSICAL activity" \| 22,649 \| \| 16 \| SU exercise* \| 153,790 \| \| 17 \| TI ( (aerobic* or aquatherap* or "aqua therap*" or bicycle* or bicycling or cycling or calisthenic* or callisthenic* or "cardiopulmonary conditioning" or climbing or danc* or exercise* or "gi gong" or gigong or gymnastic or hiit or hopping or hydrotherap* or "isometric training" or jogging or jumping or kinesiolog* or "motion therap*" or "movement therap*" or pilates or "physical condition*" or plyometric* or relaxation* or running or sport* or stretching or swim* or "tai chi" or tai or thai or taiji or taijiquan or taichi or treadmill or walk* or warm-up or "water therap*" or "weight* lifting" or "lifting weight*" or weightlifting or "power lifting" or "water therap*" or "weight training" or yoga) ) OR AB ( (aerobic* or aquatherap* or "aqua therap*" or bicycle* or bicycling or cycling or calisthenic* or callisthenic* or "cardiopulmonary conditioning" or climbing or danc* or exercise* or "gi gong" or gigong or gymnastic or hiit or hopping or hydrotherap* or "isometric training" or jogging or jumping or kinesiolog* or "motion therap*" or "movement therap*" or pilates or "physical condition*" or plyometric* or relaxation* or running or sport* or stretching or swim* or "tai chi" or tai or thai or taiji or taijiquan or taichi or treadmill or walk* or warm-up or "water therap*" or "weight* lifting" or "lifting weight*" or weightlifting or "power lifting" or "water therap*" or "weight training" or yoga) ) OR KW ( (aerobic* or aquatherap* or "aqua therap*" or bicycle* or bicycling or cycling or calisthenic* or callisthenic* or "cardiopulmonary conditioning" or climbing or danc* or exercise* or "gi gong" or gigong or gymnastic or hiit or hopping or hydrotherap* or "isometric training" or jogging or jumping or kinesiolog* or "motion therap*" or "movement therap*" or pilates or "physical condition*" or plyometric* or relaxation* or running or sport* or stretching or swim* or "tai chi" or tai or thai or taiji or taijiquan or taichi or treadmill or walk* or warm-up or "water therap*" or "weight* lifting" or "lifting weight*" or weightlifting or "power lifting" or "water therap*" or "weight training" or yoga) ) \| 696,822 \| \| 18 \| DE "SPORTS" OR DE "AERODYNAMICS in sports" OR DE "AERONAUTICAL sports" OR OR DE "AQUATIC sports" OR DE "BALL games" OR DE "BALLISTICS in sports" OR DE "BASEBALL" OR DE "COMBAT sports" OR DE "CONTACT sports" OR DE "CROSS-training (Sports)" OR DE "DISC golf" OR DE "ENDURANCE sports" OR DE "EXTREME sports" OR DE "FANTASY sports" OR DE "GYMNASTICS" OR DE "HOCKEY" OR DE "PARKOUR" OR DE "RACKET games" OR DE "RECREATIONAL sports" OR DE "ROLLER skating" OR DE "SKATEBOARDING" OR DE "SOFTBALL" OR DE "TEAM sports" \| 180,087 \| \| 19 \| TI ( physical* N2 (activ* or training) ) OR AB ( physical* N2 (activ* or training) ) OR KW ( physical* N2 (activ* or training) ) \| 65,770 \| \| 20 \| TI ( (anaerobic or cardio* or circuit or interval* or "motor control" or muscle* or resistance* or strength*) N2 (program* or training) ) OR AB ( (anaerobic or cardio* or circuit or interval* or "motor control" or muscle* or resistance* or strength*) N2 (program* or training) ) OR KW ( (anaerobic or cardio* or circuit or interval* or "motor control" or muscle* or resistance* or strength*) N2 (program* or training) ) \| 30,364 \| \| 21 \| S15 OR S16 OR S17 OR S18 OR S19 OR S20 \| 855,157 \| \| 22 \| S14 AND S21 \| 1,056 \| \| 23 \| TI ((systematic or umbrella or scoping) N2 review) \| 49,437 \| \| 24 \| TI ("meta analys*" or metaanalys* or meta synthes* or metasynthes*) \| 37,250 \| \| 25 \| TI ("review of reviews") \| 73 \| \| 26 \| S23 OR S24 OR S25 OR S26 \| 83,250 \| \| 27 \| S22 AND S26 \| 82 \| | |
