# Supplementary material for: Summarizing the effects of different exercise types in chronic neck pain – a systematic review and meta-analysis of systematic reviews
Source: BMC Musculoskelet Disord. 2023 Oct 12;24:806. doi: 10.1186/s12891-023-06930-9 (PMC10568903; doi:10.1186/s12891-023-06930-9)
Supplement: Supplementary file 4 — Additional file 4. Excluded papers based on full-text reading and reasons. [file 12891_2023_6930_MOESM4_ESM.docx]

| **Additional file 4** | | |
| --- | --- | --- |
| The following 57 papers were excluded based on full-text reading and reasons | | |
| # | Paper | EXCLUDED |
| 1 | Alagingi NK. Chronic neck pain and postural rehabilitation: A literature review. J Bodyw Mov Ther. 2022 Oct;32:201-206. doi: 10.1016/j.jbmt.2022.04.017. Epub 2022 Apr 20. PMID: 36180150. | Wrong publication |
| 2 | Alotaibi MO, Mubarak AS, Alsakhri MM, Alqurashi AK, Alshahrani NA, Felfelan II. Efficacy of Exercises on Chronic Neck Pain: A Systematic Review. International Journal of Early Childhood Special Education (INT-JECSE). doi: 10.9756/INT-JECSE/V14I2.372 ISSN:1308-5581 Vol 14, Issue 02, 2022 | Wrong intervention |
| 3 | Bayattork M, Skold MB, Sundstrup E, Andersen LL. Exercise interventions to improve postural malalignments in head, neck, and trunk among adolescents, adults, and older people: systematic review of randomized controlled trials. JOURNAL OF EXERCISE REHABILITATION 2020;16:36-48. | Wrong population |
| 4 | Binder AI. Neck pain. BMJ clinical evidence 2008. | Wrong population |
| 5 | Bonatesta L, Ruiz-Cárdenas JD, Fernández-Azorín L, Rodríguez-Juan JJ. Pain Science Education Plus Exercise Therapy in Chronic Nonspecific Spinal Pain: A Systematic Review and Meta-analyses of Randomized Clinical Trials. Journal of Pain 2022;23:535-46. | Wrong intervention |
| 6 | Byrnes K, et al. Is Pilates an effective rehabilitation tool? A systematic review. Journal of bodywork and movement therapies. 2018;22(1):192-202. | Wrong population |
| 7 | Corvillo I, Armijo F, Alvarez-Badillo A, Armijo O, Varela E, Maraver F. Efficacy of aquatic therapy for neck pain: a systematic review. International journal of biometeorology 2020;64:915-25. | Wrong publication type/no RCT |
| 8 | Coury H, Moreira RFC, Dias NB. Evaluation of the effectiveness of workplace exercise in controlling neck, shoulder and low back pain: a systematic review. BRAZILIAN JOURNAL OF PHYSICAL THERAPY 2009;13:461-79. | Wrong intervention |
| 9 | Cox LG, Kidgell DJ, Iles RA. Neck-specific strengthening exercises and cognitive therapy for chronic neck pain: a systematic review. PHYSICAL THERAPY REVIEWS 2019;24:335-45. | Wrong population |
| 10 | Crow EM, Jeannot E, Trewhela A. Effectiveness of Iyengar yoga in treating spinal (back and neck) pain: A systematic review. International journal of yoga 2015;8:3-14. | Wrong population |
| 11 | Dandale C, Telang PA, Kasatwar P. The Effectiveness of Ergonomic Training and Therapeutic Exercise in Chronic Neck Pain in Accountants in the Healthcare System: A Review. Cureus. 2023 Mar 4;15(3):e35762. doi: 10.7759/cureus.35762. PMID: 37025734; PMCID: PMC10072180. | Wrong intervention |
| 12 | Damgaard P, Bartels EM, Ris I, Christensen R, Juul-Kristensen B. Evidence of Physiotherapy Interventions for Patients with Chronic Neck Pain: A Systematic Review of Randomised Controlled Trials. ISRN Pain 2013;2013:567175. | Wrong intervention |
| 13 | Denham-Jones L, Gaskell L, Spence N, Pigott T. A systematic review of the effectiveness of Pilates on pain, disability, physical function, and quality of life in older adults with chronic musculoskeletal conditions. Musculoskeletal care 2022;20:10-30. | Wrong population |
| 14 | Denham-Jones L, Gaskell L, Spence N, Tim P. A systematic review of the effectiveness of yoga on pain, physical function, and quality of life in older adults with chronic musculoskeletal conditions. Musculoskeletal care 2022;20:47-73. | Wrong population |
| 15 | Diz JBM, de Souza J, Leopoldino AAO, Oliveira VC. Exercise, especially combined stretching and strengthening exercise, reduces myofascial pain: a systematic review. JOURNAL OF PHYSIOTHERAPY 2017;63:17-22. | Wrong population |
| 16 | Duenas L, Aguilar-Rodriguez M, Voogt L, et al. Specific versus Non-Specific Exercises for Chronic Neck or Shoulder Pain: A Systematic Review. Journal of clinical medicine 2021;10. | Wrong population |
| 17 | Ferreira GE, Barreto RGP, Robinson CC, Plentz RDM, Silva MF. Global Postural Reeducation for patients with musculoskeletal conditions: a systematic review of randomized controlled trials. BRAZILIAN JOURNAL OF PHYSICAL THERAPY 2016;20:194-205. | Wrong population |
| 18 | Fredin K, Loras H. Manual therapy, exercise therapy or combined treatment in the management of adult neck pain - A systematic review and meta-analysis. Musculoskeletal science & practice 2017;31:62-71. | Wrong intervention |
| 19 | Frutiger M, Borotkanics R. Systematic Review and Meta-Analysis Suggest Strength Training and Workplace Modifications May Reduce Neck Pain in Office Workers. Pain practice : the official journal of World Institute of Pain 2021;21:100-31. | Wrong population |
| 20 | Gross AR, Paquin JP, Blanchette S, et al. Exercise for mechanical neck disorders: A cochrane systematic review update. Physiotherapy (United Kingdom) 2015;101:eS486. | Duplicate |
| 21 | Gross A, et al. Exercises for mechanical neck disorders. The Cochrane database of systematic reviews. 2015;1:CD004250. | Duplicate |
| 22 | Heng W, Wei F, Liu Z, Yan X, Zhu K, Yang F, Du M, Zhou C, Qian J. Physical exercise improved muscle strength and pain on neck and shoulder in military pilots. Front Physiol. 2022 Sep 2;13:973304. doi: 10.3389/fphys.2022.973304. PMID: 36117716; PMCID: PMC9479108. | Wrong population |
| 23 | Hidalgo B, Hall T, Bossert J, Dugeny A, Cagnie B, Pitance L. The efficacy of manual therapy and exercise for treating non-specific neck pain: A systematic review. Journal of back and musculoskeletal rehabilitation 2017;30:1149-69. | Wrong intervention |
| 24 | Kay T, Gross A, Rutherford S, et al. Exercise therapy for neck pain: A cochrane systematic review update. Physiotherapy (United Kingdom) 2011;97:eS436-eS7. | Duplicate |
| 25 | Kay TM, Gross A, Goldsmith C, et al. Exercises for mechanical neck disorders. The Cochrane database of systematic reviews 2005:CD004250. | Duplicate |
| 26 | Kay TM, Gross A, Goldsmith CH, et al. Exercises for mechanical neck disorders. The Cochrane database of systematic reviews 2012:CD004250. | Duplicate |
| 27 | Leemans L, Polli A, Nijs J, Wideman T, den Bandt H, Beckwée D. It Hurts to Move! Intervention Effects and Assessment Methods for Movement-Evoked Pain in Patients With Musculoskeletal Pain: A Systematic Review with Meta-analysis. J Orthop Sports Phys Ther. 2022 Jun;52(6):345-374. doi: 10.2519/jospt.2022.10527. Epub 2022 Feb 5. PMID: 35128943. | Wrong intervention |
| 28 | Leaver AM, Refshauge KM, Maher CG, McAuley JH. Conservative interventions provide short-term relief for non-specific neck pain: a systematic review. Journal of physiotherapy 2010;56:73-85. | Wrong intervention |
| 29 | Lin KY, Tsai YJ, Hsu PY, Tsai CS, Kuo YL. Effects of Sling Exercise for Neck Pain: A Systematic Review and Meta-Analysis. Physical therapy 2021;101. | Wrong population |
| 30 | Liu Z, Hu H, Wen X, Liu X, Xu X, Wang Z, Li L, Liu H. Baduanjin improves neck pain and functional movement in middle-aged and elderly people: A systematic review and meta-analysis of randomized controlled trials. Front Med (Lausanne). 2023 Jan 10;9:920102. doi: 10.3389/fmed.2022.920102. PMID: 36703891; PMCID: PMC9871642. | Wrong intervention |
| 31 | Matsi AE, Atsidakou N, Christakou A, Georgoudis G. Effectiveness of craniocervical flexion exercise on pain, disability, and cervical range of motion in patients with neck pain. Critical Reviews in Physical and Rehabilitation Medicine 2020;32:39-57. | Wrong population |
| 32 | McCaskey M, Schuster-Amft C, Wenderoth N, De Bruin E. Effects of proprioceptive exercises for patients with chronic low back and neck pain: A systematic review. Annals of Physical and Rehabilitation Medicine 2014;57:e252. | Double publication |
| 33 | McCaskey MA, Schuster-Amft C, Wirth B, De Bruin ED. Effects of proprioceptive exercises on pain and function in chronic neck-and low back pain rehabilitation: A systematic literature review. Physiotherapy (United Kingdom) 2015;101:eS969-eS70. | Double publication |
| 34 | McCaskey MA, Schuster-Amft C, Wirth B, Suica Z, de Bruin ED. Effects of proprioceptive exercises on pain and function in chronic neck- and low back pain rehabilitation: a systematic literature review. BMC musculoskeletal disorders 2014;15:382. | Wrong Population |
| 35 | Mior S. Exercise in the treatment of chronic pain. The Clinical journal of pain 2001;17:S77-85. | Wrong intervention |
| 36 | Mohan V, Paungmali A, Sitilertpisan P, Henry LJ, Mohamad NB, Kharami NNB. Feldenkrais method on neck and low back pain to the type of exercises and outcome measurement tools: A systematic review. Polish Annals of Medicine 2017;24:77-83. | Wrong publication type |
| 37 | Njoku FC, Klose P, Brinkhaus B, Michalsen A, Dobos G, Cramer H. Yoga for chronic neck pain-a systematic review and meta-analysis. BMC Complementary and Alternative Medicine 2017;17. | Duplicate |
| 38 | O'Keeffe M, Hayes A, McCreesh K, Purtill H, O'Sullivan K. The comparative effectiveness of exercise-based group and individual physiotherapy for musculoskeletal conditions: A systematic review and meta-analysis. Manual Therapy 2016;25:e122. | Duplicate |
| 39 | O'Keeffe M, Hayes A, McCreesh K, Purtill H, O'Sullivan K. Are group-based and individual physiotherapy exercise programmes equally effective for musculoskeletal conditions? A systematic review and meta-analysis. British journal of sports medicine 2017;51:126-32. | Wrong population |
| 40 | O'Riordan C, Clifford A, Van De Ven P, Nelson J. Chronic neck pain and exercise interventions: frequency, intensity, time, and type principle. Archives of physical medicine and rehabilitation 2014;95:770-83. | Wrong intervention |
| 41 | Paraskevopoulos E, Koumantakis GA, Papandreou M. A Systematic Review of the Aerobic Exercise Program Variables for Patients with Non-Specific Neck Pain: Effectiveness and Clinical Applications. Healthcare (Basel). 2023 Jan 24;11(3):339. doi: 10.3390/healthcare11030339. PMID: 36766914; PMCID: PMC9914281. | Wrong population |
| 42 | Price J, et al. Effectiveness and optimal dosage of resistance training for chronic neck pain: a systematic review with a qualitative synthesis and meta-analysis. Physiotherapy (United Kingdom). 2020;107:e48. | Double publication |
| 43 | Sheikhhoseini R, Shahrbanian S, Sayyadi P, O'Sullivan K. Effectiveness of Therapeutic Exercise on Forward Head Posture: A Systematic Review and Meta-analysis. Journal of manipulative and physiological therapeutics 2018;41:530-9. | Wrong population |
| 44 | Sihawong R, Janwantanakul P, Sitthipornvorakul E, Pensri P. Exercise therapy for office workers with non-specific neck pain: Systematic review. Physiotherapy (United Kingdom) 2011;97:eS551-eS2. | Wrong intervention |
| 45 | Taylor FR, Landy SH, Kaniecki RG. Miller J, Gross A, D'Sylva J, Burnie SJ, Goldsmith CH, Graham N, Haines T, Brønfort G, Hoving JL. Manual therapy and exercise for neck pain: a systematic review. Man Ther. 2010;15:334-354. Headache: The Journal of Head & Face Pain 2010;50:1623-. | Wrong population |
| 46 | Verhagen AP, Karels C, Bierma-Zeinstra SMA, et al. Ergonomic and physiotherapeutic interventions for treating work-related complaints of the arm, neck or shoulder in adults. COCHRANE DATABASE OF SYSTEMATIC REVIEWS 2006:CD003471. | Publication withdrawn |
| 47 | Visvanathan R, Paul J, Manoharlal MA, Muthuswamy S, Muthukumar N. Efficacy of endurance exercise on pain and disability in chronic neck pain-a systematic review. Journal of Clinical and Diagnostic Research 2018;12:5-13. | Wrong population |
| 48 | Wilhelm MP, Donaldson M, Griswold D, et al. The Effects of Exercise Dosage on Neck-Related Pain and Disability: A Systematic Review With Meta-analysis. The Journal of orthopaedic and sports physical therapy 2020;50:607-21. | Wrong intervention |
| 49 | Wu B, Yuan H, Geng D, Zhang L, Zhang C. The Impact of a Stabilization Exercise on Neck Pain: A Systematic Review and Meta-analysis. Journal of neurological surgery Part A, Central European neurosurgery 2020;81:342-7. | Wrong intervention |
| 50 | Yamato TP, Saragiotto BT, Maher C. Therapeutic exercise for chronic non-specific neck pain: PEDro systematic review update. British journal of sports medicine 2015;49:1350. | Duplicate |
| 51 | Ylinen J. Physical exercises and functional rehabilitation for the management of chronic neck pain. Europa medicophysica 2007;43:119-32. | Wrong intervention |
| 52 | Zacharakis AM, Zanelli LM, Watkins HR, et al. What is the Evidence for the Effectiveness of Scapulothoracic Strengthening Exercises in Individuals with Neck Pain? A Systematic Review. INTERNET JOURNAL OF ALLIED HEALTH SCIENCES AND PRACTICE 2020;18. | Wrong population |
| 53 | Zang C, Zhou YY, Liu YH, Wu BB. Efficacy of Stabilization Exercise for Neck Pain: A Narrative Review and Meta-Analysis of Randomized Controlled Studies. PHYSIKALISCHE MEDIZIN REHABILITATIONSMEDIZIN KURORTMEDIZIN 2020;30:26-32. | Wrong population |
| 54 | Zhou J, Yu Y, Cao B, et al. Characteristic of Clinical Studies on Baduanjin during 2000-2019: A Comprehensive Review. Evidence-based Complementary and Alternative Medicine 2020;2020:4783915. | Wrong population |
| 55 | Zou LY, Pan ZJ, Yeung A, et al. A Review Study on the Beneficial Effects of Baduanjin. JOURNAL OF ALTERNATIVE AND COMPLEMENTARY MEDICINE 2018;24:324-35. | Wrong population |
| 56 | Zou LY, Yeung A, Quan XF, Boyden SD, Wang HR. A Systematic Review and Meta-Analysis of Mindfulness-Based (Baduanjin) Exercise for Alleviating Musculoskeletal Pain and Improving Sleep Quality in People with Chronic Diseases. INTERNATIONAL JOURNAL OF ENVIRONMENTAL RESEARCH AND PUBLIC HEALTH 2018;15. | Wrong population |
| 57 | Zronek M, Sanker H, Newcomb J, Donaldson M. The influence of home exercise programs for patients with non-specific or specific neck pain: a systematic review of the literature. The Journal of manual & manipulative therapy 2016;24:62-73. | Wrong population |
