# Supplementary material for: Summarizing the effects of different exercise types in chronic neck pain – a systematic review and meta-analysis of systematic reviews
Source: BMC Musculoskelet Disord. 2023 Oct 12;24:806. doi: 10.1186/s12891-023-06930-9 (PMC10568903; doi:10.1186/s12891-023-06930-9)

### Additional file 5

### **Meta-analyses based on data from the included SRs (MAs) for each exercise type**

### Forest plots for the effect sizes of specific exercise types for pain intensity, disability and for short-term and intermediate/long-term. The plot depicts model fit, individual study and pooled effect size estimates (standardized mean differences and corresponding 95% confidence intervals), separated for the different comparators (non-exercise controls and exercise controls). The size of the boxes corresponds to the respective studies’ (inverse variance) weighting. SMD: standardized mean difference; CI: confidence interval. SMDs were calculated when at least two SRs provided data.

**5.1 MCE: Outcome pain-intensity: MCE compared to non-exercise controls, short-term**

**
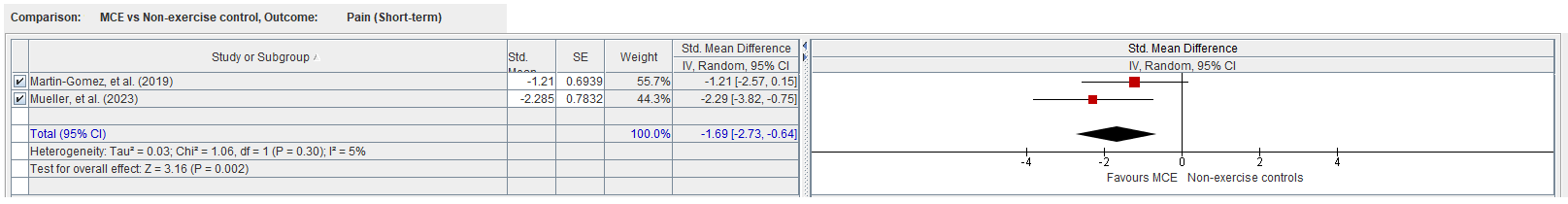
**

**5.2 MCE: Outcome pain-intensity: MCE compared to exercise controls, short-term**

**
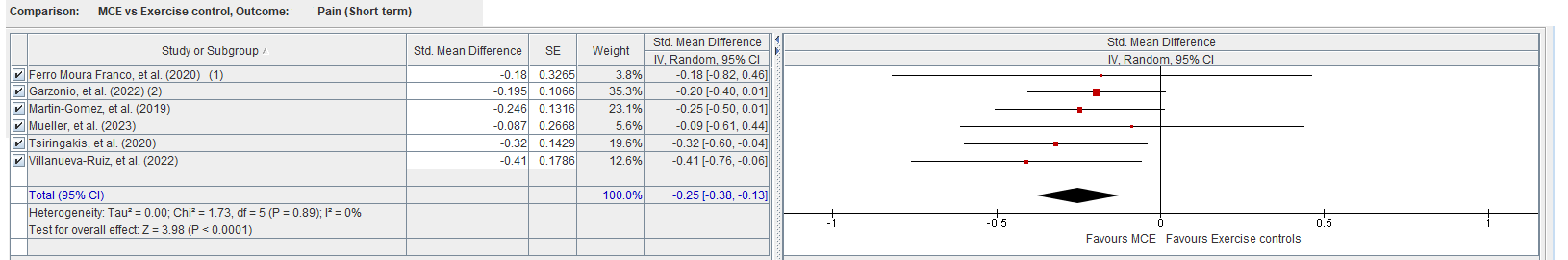
**

**5.3 MCE: Outcome disability: MCE compared to non-exercise controls, short-term**

**
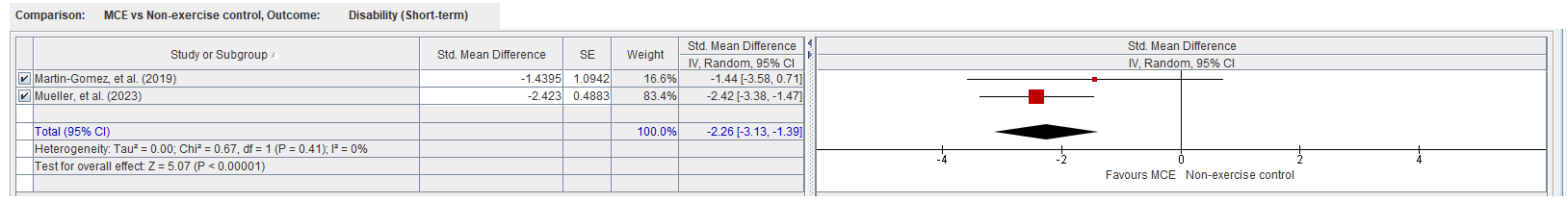
**

**5.4 MCE: Outcome disability: MCE compared to exercise controls, short-term**

**
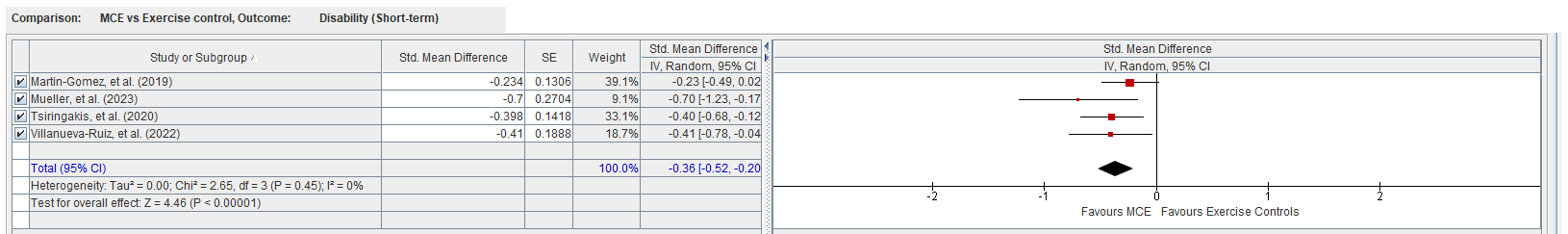
**

**5.5 Resistance training: Outcome pain-intensity: Resistance exercises compared to non-exercise controls, short-term**

**
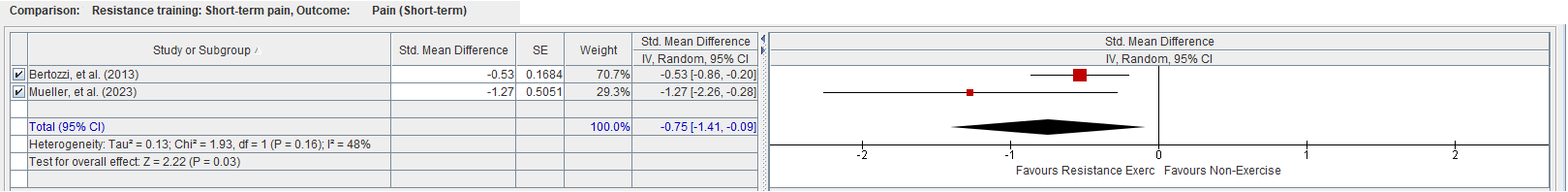
**

**5.6 Resistance training: Outcome pain-intensity: Resistance exercises compared to non-exercise controls, intermediate/long-term**


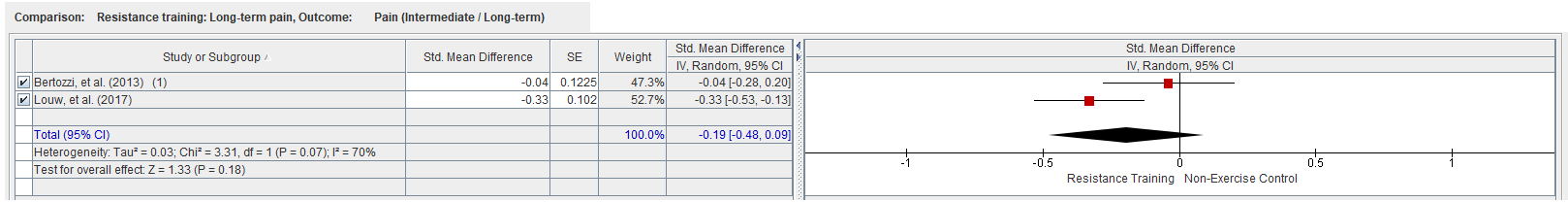


**5.7 Resistance training: Outcome pain-intensity: Resistance exercises compared to Exercise controls, short-term**


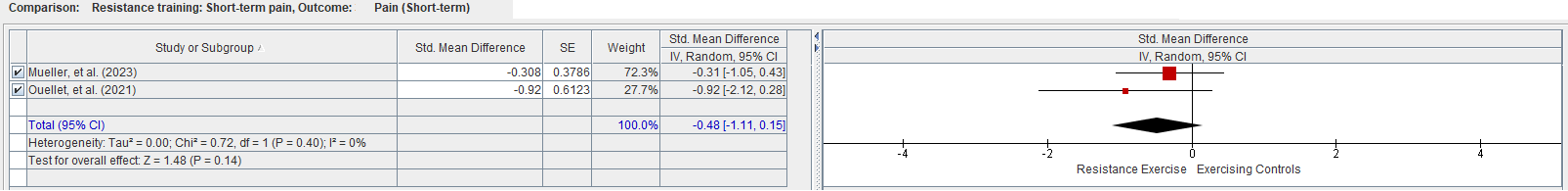


**5.8 Resistance training: Outcome disability: Resistance exercises compared to non-exercise controls, short-term**


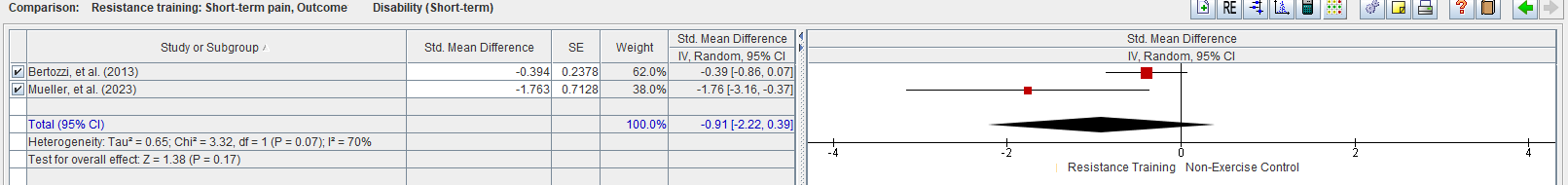


**5.9 Resistance training: Outcome disability compared to non-exercise controls, intermediate/long-term**


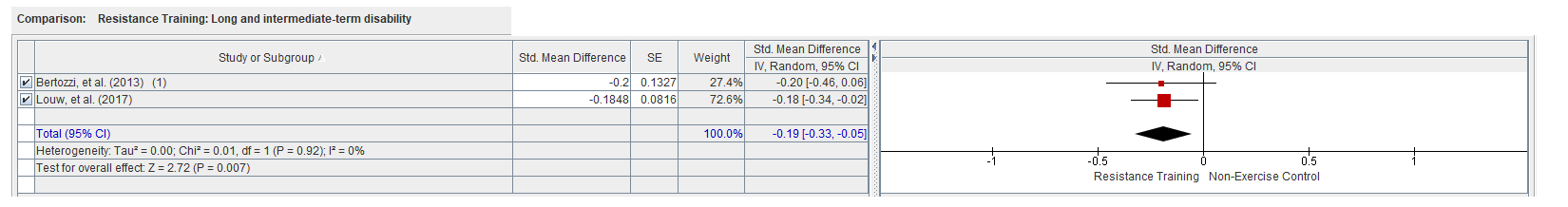


**5.10 TCE: Outcome pain-intensity: TCE compared to non-exercise controls, short-term**


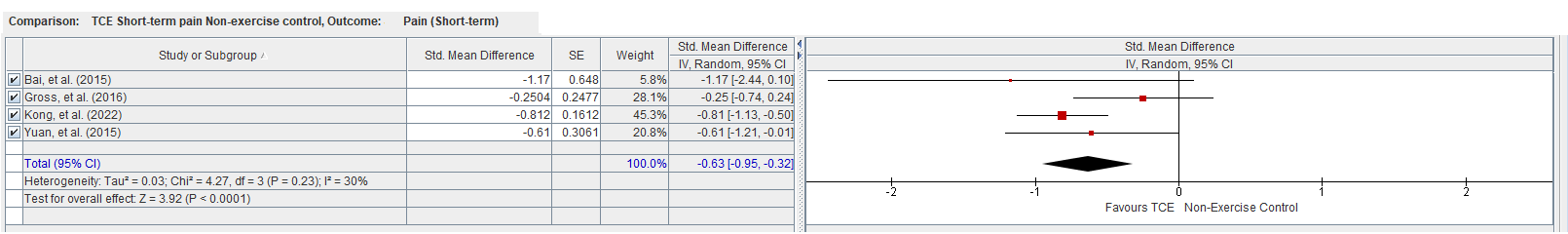


**5.11 TCE: Outcome pain-intensity: TCE compared to non-exercise controls, intermediate/long-term**


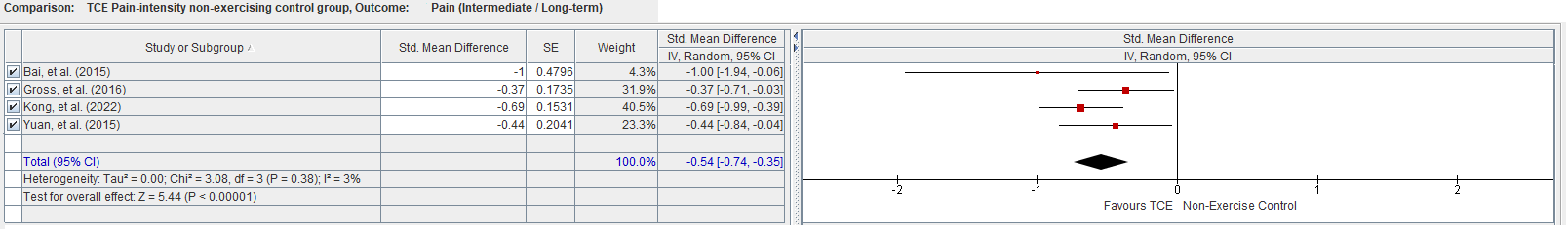


**5.12 TCE: Outcome pain-intensity: TCE compared to exercise controls, short-term**


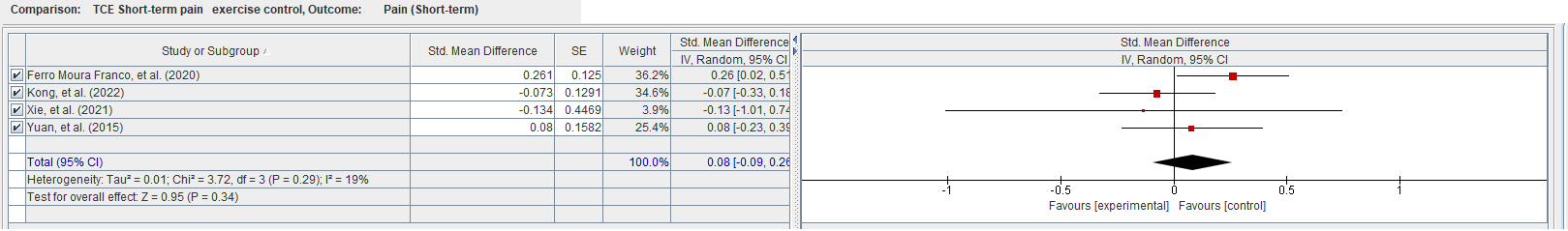


**5.13 TCE: Outcome disability: TCE compared to non-exercise controls, short-term**


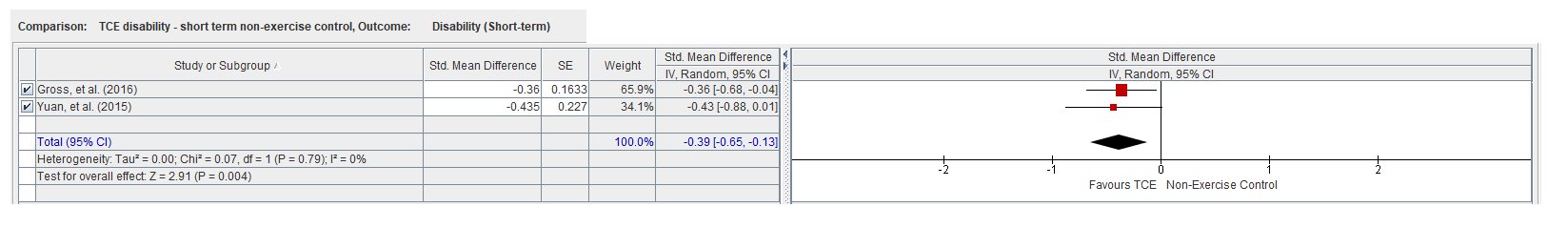


**5.14 TCE: Outcome disability: TCE compared to non-exercise controls, intermediate/long-term**


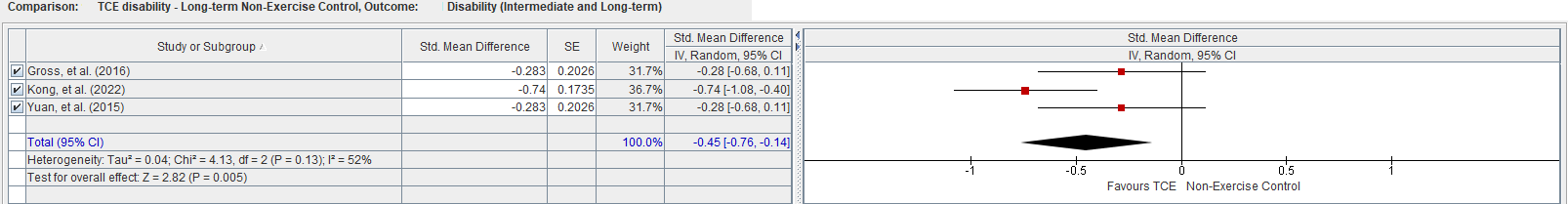


**5.15 TCE: Outcome disability: TCE compared to exercise controls, short-term**


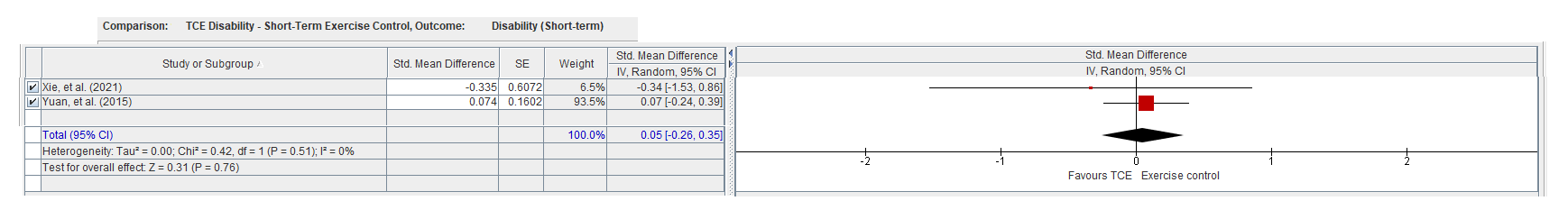


**5.16 Yoga: Outcome pain-intensity: Yoga compared to non-exercise controls, short-term**


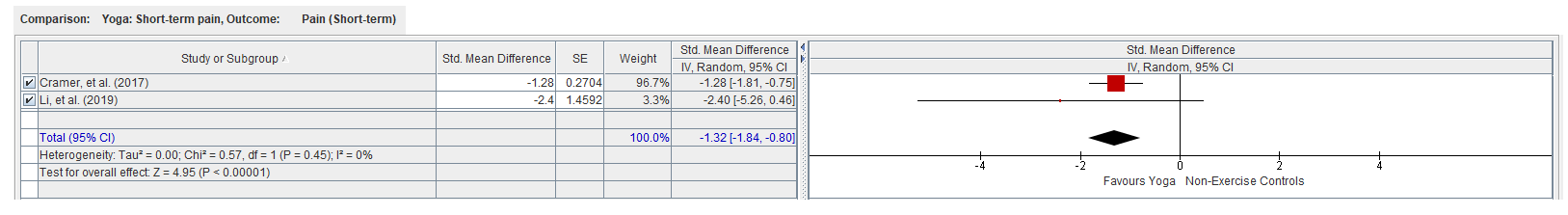


**5.17 Yoga: Outcome disability: Yoga compared to non-exercise controls, short-term**


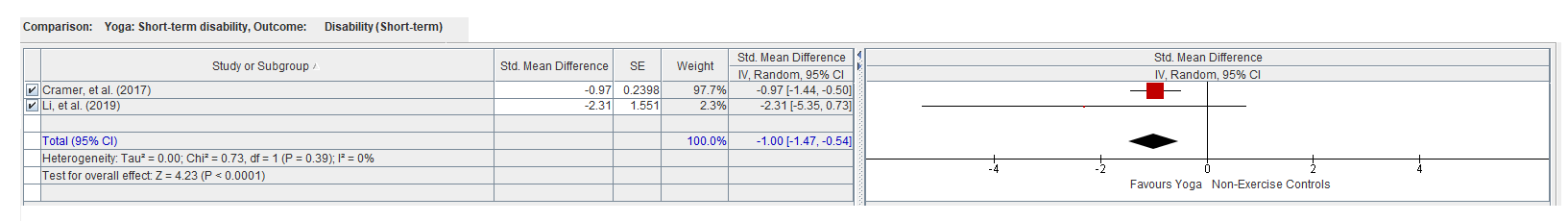

Supplement: Supplementary file 5 — Additional file 5. Meta-analyses based on data from the included SRs (MAs) for each exercise type. [file 12891_2023_6930_MOESM5_ESM.docx]
